# Defects in 8-oxo-guanine repair pathway cause high frequency of C > A substitutions in neuroblastoma

**DOI:** 10.1073/pnas.2007898118

**Published:** 2021-09-03

**Authors:** Marlinde L. van den Boogaard, Rurika Oka, Anne Hakkert, Linda Schild, Marli E. Ebus, Michael R. van Gerven, Danny A. Zwijnenburg, Piet Molenaar, Lieke L. Hoyng, M. Emmy M. Dolman, Anke H. W. Essing, Bianca Koopmans, Thomas Helleday, Jarno Drost, Ruben van Boxtel, Rogier Versteeg, Jan Koster, Jan J. Molenaar

**Affiliations:** ^a^Princess Máxima Center for Pediatric Oncology, 3584 CS, Utrecht, The Netherlands;; ^b^Oncode Institute, Princess Máxima Center for Pediatric Oncology, 3584 CS, Utrecht, The Netherlands;; ^c^Department of Oncogenomics, Academic Medical Center, 1105 AZ, Amsterdam, The Netherlands;; ^d^Science for Life Laboratory, Department of Oncology–Pathology, Karolinska Institutet, S-171 76 Stockholm, Sweden;; ^e^Weston Park Cancer Centre, Department of Oncology and Metabolism, University of Sheffield, S10 2RX Sheffield, United Kingdom

**Keywords:** neuroblastoma, 8-oxo-guanine repair, MUTYH, OGG1, mutational signatures

## Abstract

Neuroblastomas are childhood tumors with frequent fatal relapses after induction treatment, which is related to tumor evolution with additional genomic events. Our whole-genome sequencing data analysis revealed a high frequency of somatic cytosine > adenine (C > A) substitutions in primary neuroblastoma tumors, which was associated with poor survival. We showed that increased levels of C > A substitutions correlate with copy number loss (CNL) of *OGG1* or *MUTYH*. Both genes encode DNA glycosylases that recognize 8-oxo-guanine (8-oxoG) lesions as a first step of 8-oxoG repair. Tumor organoid models with CNL of *OGG1* or *MUTYH* show increased 8-oxoG levels compared to wild-type cells. We used CRISPR-Cas9 genome editing to create knockout clones of *MUTYH* and *OGG1* in neuroblastoma cells. Whole-genome sequencing of single-cell *OGG1* and *MUTYH* knockout clones identified an increased accumulation of C > A substitutions. Mutational signature analysis of these *OGG1* and *MUTYH* knockout clones revealed enrichment for C > A signatures 18 and 36, respectively. Clustering analysis showed that the knockout clones group together with tumors containing *OGG1* or *MUTYH* CNL. In conclusion, we demonstrate that defects in 8-oxoG repair cause accumulation of C > A substitutions in neuroblastoma, which contributes to mutagenesis and tumor evolution.

## Significance

The collection of large amounts of whole-genome sequencing data allowed for identification of mutational signatures, which are characteristic combinations of substitutions in the context of neighboring bases. The clinical significance of these mutational signatures is still largely unknown. In neuroblastoma, we showed that high levels of cytosine > adenine (C > A) substitutions are associated with poor survival. We identified that these high levels of C > A substitutions result from defects in 8-oxo-guanine repair, specifically from copy number loss of the DNA glycosylases *MUTYH* and *OGG1*. The high frequency of C > A substitutions in neuroblastoma contributes to the increased adaptive capacity of these tumors. Thereby, we link basic molecular genetic mutation patterns to clinically significant tumor evolution processes.

Neuroblastoma is a childhood tumor of the peripheral sympathetic nervous system that accounts for 15% of childhood cancer mortality (1). Survival rates vary between spontaneous remissions in the low-stage neuroblastoma tumors to less than 50% survival in the high-stage patient group (2). Like other pediatric cancer types, the number of somatic mutations in neuroblastoma is relatively low compared to adult cancers (3). Relapsed neuroblastoma tumors have an increased mutational burden compared to primary tumors and show recurrent alterations in, e.g., the RAS-MAPK pathway (4, 5).

**Fig. 1.**
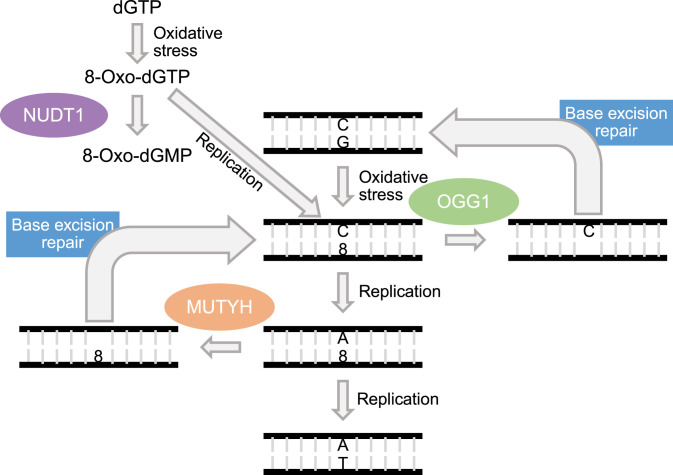
Schematic overview of 8-oxoG repair pathway. When a G in the DNA is oxidized to an 8-oxo-guanine [8-oxoG(8)], the replicative DNA polymerases will insert an A opposite of the 8-oxoG during replication. In the next round of replication, a T will be inserted opposite of the A, resulting in a C > A substitution. The DNA glycosylases *OGG1* and *MUTYH* are able to recognize 8-oxoG base pairing with a C or A, respectively. *OGG1* excises 8-oxoG from the DNA, while *MUTYH* excises the A opposite of the 8-oxoG. The DNA is further repaired by the base excision repair pathway to a C:G base pair for *OGG1*-initiated repair or to a C:8-oxoG for *MUTYH*-initiated repair. This C:8-oxoG base pair is then again a substrate for *OGG1*. In addition, *NUDT1* prevents the incorporation of 8-oxo-dGTP into the DNA, by hydrolyzing 8-oxo-dGTP, from the free nucleotide pool, to 8-oxo-dGMP.

Over the last years, substitution patterns and mutational signatures have been investigated in adult and pediatric cancer types (3, 6, 7). These mutational signatures are characteristic combinations of substitutions in the context of neighboring bases (6). For some of these mutational signatures, the underlying mutational process has been described, while it is unknown for others (6, 8). In the majority of pediatric cancer types, cytosine > thymine substitutions are most abundant, which has been linked to spontaneous deamination of 5-methyl-cytosines and mutational signature 1 (3). Several studies reported a higher frequency of cytosine > adenine (C > A) substitutions in neuroblastoma (3, 7). C > A mutations can result from defects in 8-oxo-guanine (8-oxoG) repair (9). 8-oxoG is one of the most abundant DNA lesions generated by reactive oxygen species (10–12). 8-oxoG can base pair with both a cytosine (C) and an adenine (A). During replication, DNA polymerases can therefore insert an incorrect A opposite of 8-oxoG. In the next round of replication, a thymine (T) will be inserted opposite the A, finally resulting in a C > A substitution (13, 14). To combat reactive oxygen species–induced DNA damage, 8-oxoG can be recognized by the DNA glycosylases *OGG1* and *MUTYH* when it forms a base pair with a C or A, respectively. 8-oxoG repair is initiated by excision of 8-oxoG by *OGG1* or excision of the wrongly inserted A opposite 8-oxoG by *MUTYH* (Fig. 1). In addition, *NUDT1* prevents incorporation of 8-oxo-dGTP from the free nucleotide pool, by hydrolysis of 8-oxo-dGTP to 8-oxo-GMP, which cannot be incorporated (13, 15, 16).

In this study, we identify increased accumulation of C > A substitutions in high-risk neuroblastoma, resulting in a strong contribution of mutational signature 18 and 36 in these tumors. We show that neuroblastoma tumors with high C > A substitution frequencies were enriched for copy number loss (CNL) of *OGG1* and *MUTYH*. To mimic this phenotype, we used CRISPR-CAS9 to engineer defects in the 8-oxoG repair genes *OGG1* and *MUTYH* in neuroblastoma cells, resulting in an increased accumulation of C > A substitutions in single-cell knockout clones and a high contribution of C > A mutational signatures 18 and 36. In clustering analysis, these clones group together with neuroblastoma tumors with *OGG1* or *MUTYH* CNL. Finally, we evaluated a neuroblastoma relapse cohort and identified that 47% of alterations in RAS-MAPK pathway genes were caused by C > A substitutions. Taken together, our study identifies that defects in the 8-oxoG repair pathway cause an accumulation of C > A substitutions in neuroblastoma tumors, potentially leading to increased adaptive capacity and tumor evolution.

## Results

### High Frequency of C > A Substitutions Correlate with Poor Prognosis in Neuroblastoma.

**Fig. 2.**
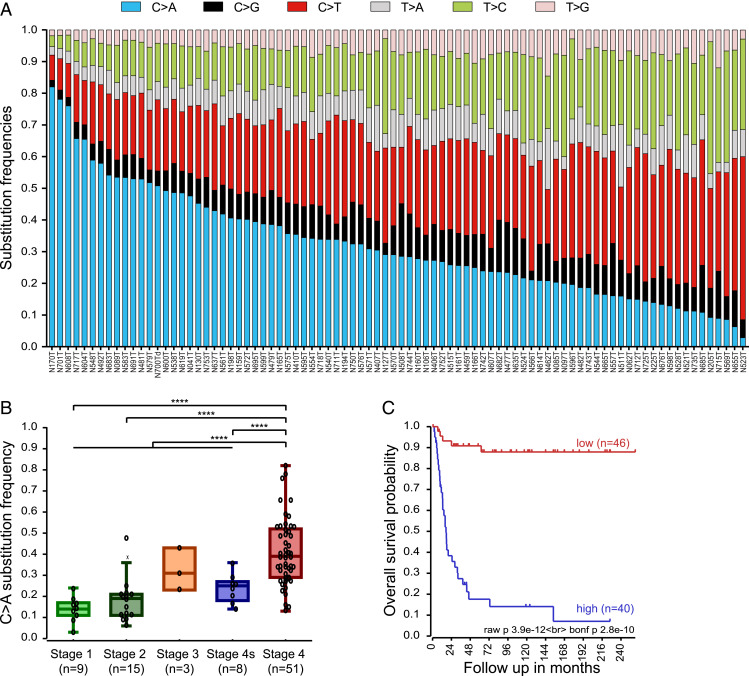
High percentages of C > A substitutions in neuroblastoma are correlated with high-stage disease and poor prognosis. (*A*) Relative substitution frequency of the six possible nucleotide substitutions, indicated with different colors, in a cohort of 86 neuroblastoma tumors. Tumors are ranked from left to right on C > A substitution frequency. (*B*) Dot-boxplot presenting the C > A substitution frequency for the different stages of neuroblastoma (INSS, st1 = stage1, st2 = stage2, st3 = stage 3, st4 = stage 4, st4s = stage 4s) in the same cohort. C > A substitution frequency is significantly higher (unpaired *t* test with Welch correction, two-sided) in stage 4 neuroblastoma compared to lower stages. ****P < 0.0001. The central line in the boxplot indicates the median and the box limits indicate the first and third quartile. The whiskers denote the interval within 1.5 times the interquartile range of the median. Outliers are indicated with a cross. The number of patients per stage is indicated below the graph. (*C*) Kaplan–Meier curve was created using Kaplan scanning for the optimal curves. This curve indicates a significantly worse survival for patients with high compared to low C > A substitution frequency (Bonferroni-corrected *P* = 2.8 × 10^−10^).

We determined the somatic frequency of each of the six possible base substitutions in our whole-genome sequencing (WGS) data of 86 primary neuroblastoma tumor/normal pairs (17). C > A substitutions were the most abundant base substitution type, including 14 tumors with a C > A substitution frequency higher than 50% (Fig. 2*A* and *SI Appendix*, Fig. S1 *A* and *B*).

Since C > A substitutions have been suggested to also occur from sequencing artifacts (18), we reanalyzed available Sanger sequencing results to confirm the C > A substitutions on the original tumor biopsies (17). Sanger sequencing validation of C > A substitutions was similar to validation of other substitutions (*SI Appendix*, Fig. S1*C*). In addition, C > A ratios were not enriched in the control DNA (leukocytes) (*SI Appendix*, Fig. S1*D*). Finally, similar levels of C > A substitutions were found in tumors and their corresponding organoid cell lines (*SI Appendix*, Fig. S1*E*). Therefore, we conclude that the C > A substitutions found in our tumor cohort were authentic.

We next compared the C > A substitution frequencies for the different tumor stages and identified significantly more C > A substitutions in International Neuroblastoma Staging System (INSS) stage 4 tumors compared to the low-stage tumors (unpaired *t* test with Welch correction, two-sided, *P* < 0.0001; Fig. 2*B*). Furthermore, a Kaplan-Meier analysis indicates a significant logrank (*P* = 2.8 × 10^−10^) difference in overall survival between patients with a high and a low percentage of C > A substitutions (Fig. 2*C*), independent of the prognostic factors of age, MYCN status, and INSS stage (*SI Appendix*, Table S1). These results show that the frequency of C > A substitutions is strongly increased in neuroblastoma tumors and that this correlates with a poor prognosis.

### High Levels of C > A Substitutions Correlate with Defects in *MUTYH* and *OGG1*.

**Fig. 3.**
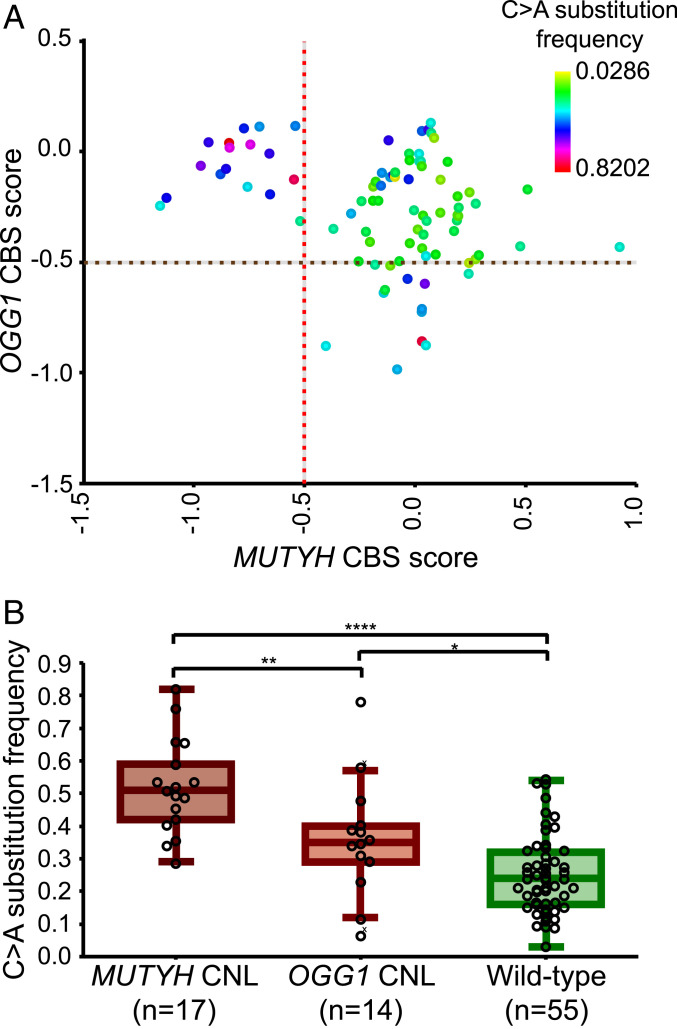
High frequency of C > A substitutions correlates with loss of *MUTYH* or *OGG1*. (*A*) The circular binary segmentation (CBS) scores for *MUTYH* and *OGG1* are plotted for 86 neuroblastoma tumors. The dots represent individual tumor samples and are colored for relative C > A substitution frequency as indicated in the color scale. The red and brown dashed line indicate the cutoff (CBS < −0.5) for *MUTYH* CNL and *OGG1* CNL, respectively. (*B*) Dot-boxplot presenting the C > A substitution frequency for neuroblastoma tumors with *MUTYH* CNL (CBS < −0.5), *OGG1* CNL (CBS < −0.5), or wild-type (MUTYH and OGG1 CBS > −0.5). The number of tumors per group is indicated below the graph. C > A substitution frequency is significantly higher (unpaired *t* test with Welch correction, two-sided) in neuroblastoma tumors with *MUTYH* CNL or *OGG1* CNL compared to wild-type. ****P < 0.0001, *P < 0.05. The central line in the boxplot indicates the median and the box limits indicate the first and third quartile. The whiskers denote the interval within 1.5 times the interquartile range of the median. Outliers are indicated with a cross.

C > A substitutions can result from defects in the 8-oxoG repair pathway (9). Therefore, we analyzed WGS data of our neuroblastoma cohort for CNL and mutation status of *MUTYH*, *OGG1*, and *NUDT1*. In a subset of tumors, we could identify CNL of *MUTYH* or *OGG1* (*SI Appendix*, Fig. S2 *A* and *B*). *OGG1* is located on 3p.25, which is part of the frequently deleted 3p region in neuroblastoma. *MUTYH*, which is located on 1p.34, was deleted in a subset of tumors with (partial) 1p CNL. *NUDT1* CNL was identified in two tumors, which also had a *MUTYH* CNL (*SI Appendix*, Table S2). CNL of *OGG1* and *MUTYH* was associated with a high frequency of C > A substitutions (Fig. 3*A*). Compared to tumors with a normal copy number of *OGG1* or *MUTYH*, a significantly higher C > A substitution frequency is detected in tumors with CNL of *MUTYH* (unpaired *t* test with Welch correction, two-sided, *P* < 0.0001) or *OGG1* (unpaired *t* test with Welch correction, two-sided, *P* = 0.0418; Fig. 3*B*). The tumor (N170T) with the highest C > A substitution frequency (82%) carried a germline missense variant in *MUTYH* (NP_036354.1:p.Ala425Pro) and a CNL of the other *MUTYH* allele (*SI Appendix*, Table S2). This missense variant in N170T is not reported in the Single Nucleotide Polymorphism Database (dbSNP) (build 153). In addition, this mutation results in an amino acid change from an alanine to a proline, which may affect the protein conformation, although PolyPhen and SIFT do not predict the variant to be damaging. Another tumor (N701T) with a very high frequency (78%) of C > A substitutions carried a germline missense variant in *OGG1* (NP_002533.1:p.Gly308Glu) and a CNL of the other *OGG1* allele (*SI Appendix*, Table S2). For this missense variant, it is predicted that it affects function. Overall, five of the seven tumors with the highest C > A substitution frequencies have multiple alterations in genes of the 8-oxoG repair pathway (*SI Appendix*, Table S2). These results show that C > A substitution frequencies are increased in neuroblastoma tumors with *OGG1* or *MUTYH* CNL.

### 8-oxoG Levels Are Increased in Cell Lines with Defects in *MUTYH* and *OGG1*.

**Fig. 4.**
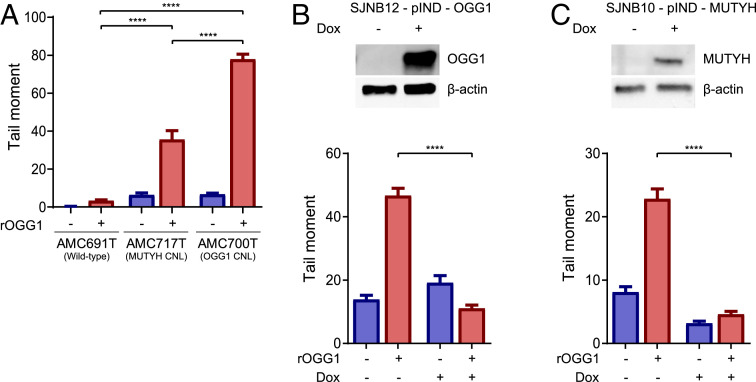
Defects in the 8-oxoG repair pathway result in higher 8-oxoG levels. Tail moments were calculated from modified comet assays with [red bar (+)] or without [blue bar (−)] recombinant *OGG1* (rOGG1) treatment. (*A*) Modified comet assays were performed for two organoids with defects in the 8-oxoG pathway (AMC717T with *MUTYH* CNL, AMC700T with *OGG1* CNL) and one organoid with a functional 8-oxoG pathway (AMC691T). (*B*, *Upper*) *OGG1* protein expression in SJNB12 (with *OGG1* CNL) transduced with a pIND-OGG1 vector with (+) or without (−) addition of doxycycline (Dox). β-actin is used as a loading control. (*Lower*) Tail moments calculated from modified comet assays for SJNB12 with (+) or without (−) Dox-induced *OGG1* overexpression. (*C*, *Upper*) *MUTYH* protein expression in SJNB10 (with *MUTYH* CNL) transduced with a pIND-MUTYH + or − Dox. β-actin is used as a loading control. (*Lower*) Tail moments calculated from modified comet assays for SJNB10 with (+) or without (−) Dox-induced *MUTYH* overexpression. Error bars indicate the SEM. *P* values are calculated with unpaired *t* test with Welch correction, two-sided, ****P < 0.0001, **P < 0.01, *P < 0.05.

To study the relation between 8-oxoG levels and defects in the 8-oxoG repair pathway, we used modified alkaline comet assays (19). In this assay, cells embedded in agarose are lysed and treated with purified recombinant OGG1, which creates DNA nicks where 8-oxoG is present. Subsequent gel electrophoresis will separate the nicked DNA from undamaged DNA. With this assay, the DNA can be visualized as comets (undamaged DNA) and tails (nicked DNA). The average tail moment can be used as a measure for the induced DNA damage and therefore for the amount of 8-oxoG. For some of the tumors, we have established tumor-derived organoid cultures (20), and similar C > A substitution percentages were identified in the organoids compared to the corresponding tumor (*SI Appendix*, Fig. S1*D*). Compared to a wild-type organoid, two organoids with a defect in the 8-oxoG repair pathway show significantly longer comet tails (unpaired *t* test with Welch correction, two-sided, *P* < 0.0001), indicative of higher levels of 8-oxoG (Fig. 4*A* and *SI Appendix*, Fig. S3*A*). To show that the increased levels of 8-oxoG are indeed caused by the defects in *OGG1* and *MUTYH*, we performed rescue experiments by inducing overexpression of wild-type OGG1 or MUTYH in neuroblastoma cell lines with CNL of *OGG1* or *MUTYH*, respectively. Indeed, we could rescue high levels of 8-oxoG by overexpression (72 h) of wild-type OGG1 or MUTYH, as indicated by a significant decrease in comet tail size (unpaired *t* test with Welch correction, *P* < 0.0001; Fig. 4 *B* and *C* and *SI Appendix*, Fig. S3 *B* and *C*). In a neuroblastoma tumor (N701T) with one of the highest C > A mutation frequencies, a germline *OGG1* missense variant was identified, as mentioned above. To test whether this mutation in *OGG1* is indeed inactivating, we also induced overexpression of *OGG1* containing the missense variant (p.G308E) in the neuroblastoma cell line with *OGG1* CNL. Since overexpression of this variant increases the tail moment, this shows that this variant does not rescue the levels of 8-oxoG (*SI Appendix*, Fig. S4 *A* and *B*), confirming that this is an inactivating event in OGG1. In addition, we also tested the effect of a germline *MUTYH* missense variant identified in the tumor with the highest C > A substitution frequency (N170T). Induced overexpression of *MUTYH* containing this missense variant (p.A425P) in the neuroblastoma cell line with *MUTYH* CNL, resulted in a lower tail moment compared to the uninduced control (*SI Appendix*, Fig. S4 *C* and *D*). Nevertheless, the tail moment remains higher than for induced overexpression without recombinant *OGG1*. This indicates that this variant does not rescue the levels of 8-oxoG to a similar extent as wild-type MUTYH and suggests that this variant reduces *MUTYH* activity. Overall, these results indicate that 8-oxoG levels are increased in neuroblastoma organoids and cell lines with defects in *MUTYH* and *OGG1* and that this can be rescued by overexpression of the affected wild-type gene.

### Increased Accumulation of C > A Substitutions upon *OGG1* or *MUTYH* Knockout in Neuroblastoma Cell Line.

**Fig. 5.**
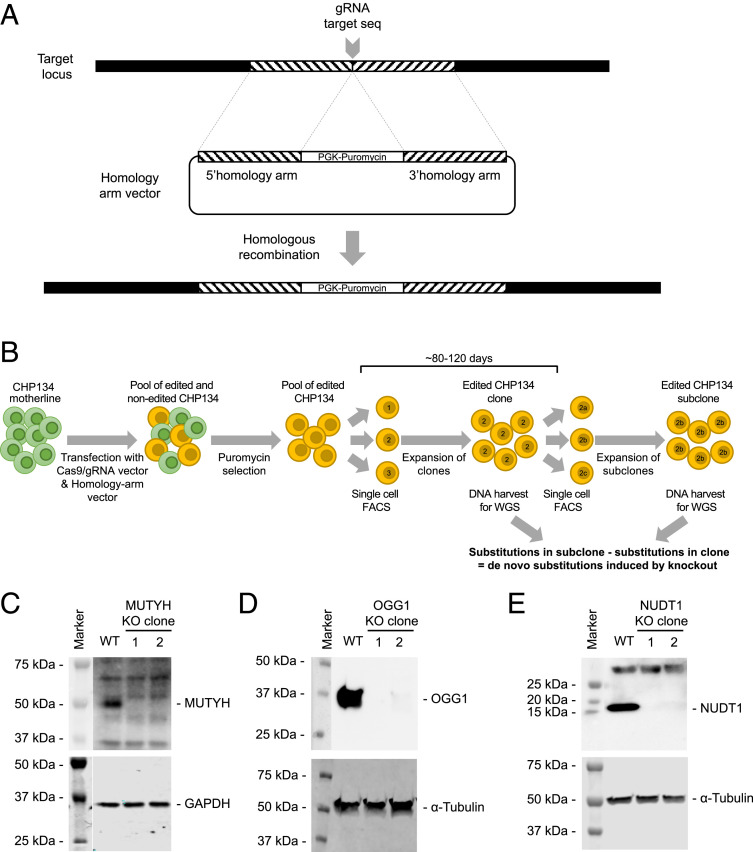
CRISPR-Cas9 editing and clonal selection of *MUTYH*, *OGG1*, and *NUDT1* knockout (sub)clones in CHP134 cell line. (*A*) CRISPR-Cas9 editing strategy used for targeting *MUTYH*, *OGG1*, or *NUDT1* in CHP134. (*B*) Schematic overview of the strategy used for creating clones and subclones of CRISPR-Cas9–edited and wild-type CHP134. Both clones and subclones were sent for WGS to evaluate the mutations that accumulate during clonal outgrowth. (*C*) Western blot analysis of *MUTYH* expression in wild-type and selected *MUTYH* knockout clones of CHP134. GAPDH is used as a loading control. (*D*) Western blot analysis of *OGG1* expression in wild-type and selected *OGG1* knockout clones of CHP134. α-tubulin is used as a loading control. (*E*) Western blot analysis of *NUDT1* expression in wild-type and selected *NUDT1* knockout clones of CHP134. α-tubulin is used as a loading control.

**Fig. 6.**
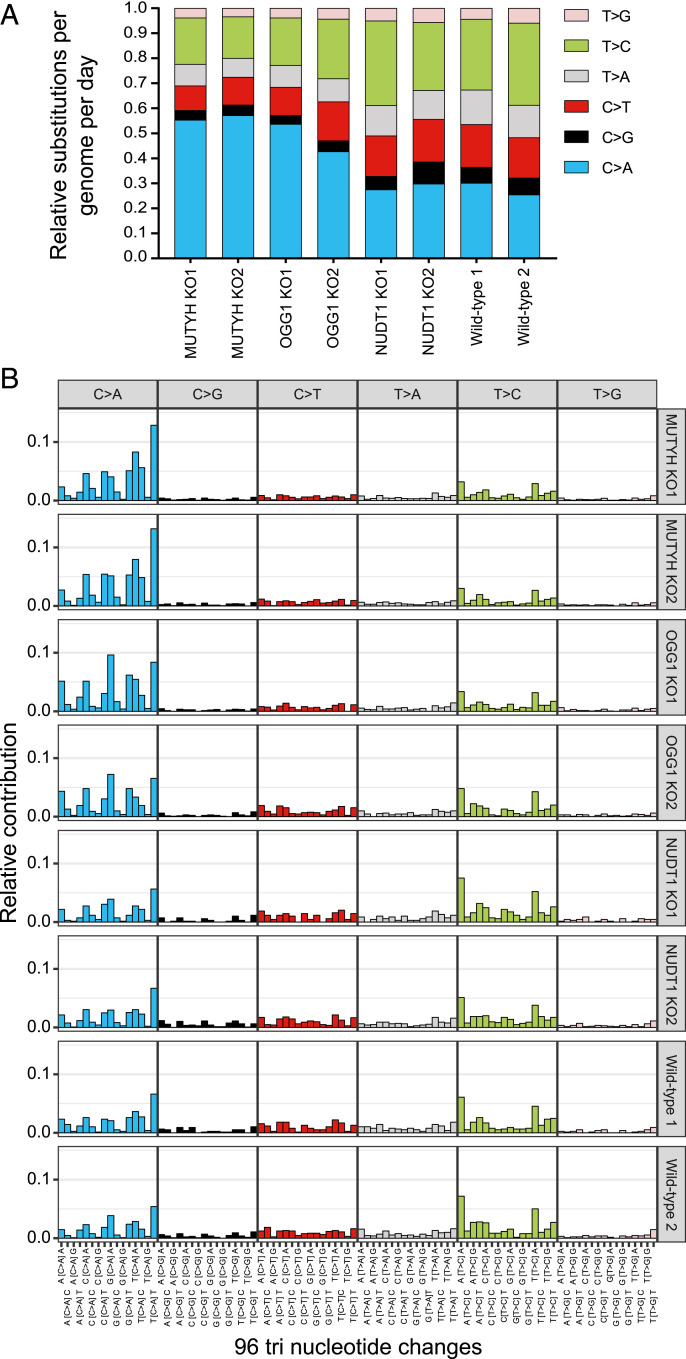
*MUTYH* or *OGG1* knockout results in accumulation of C > A substitutions in neuroblastoma cell line. (*A*) Relative substitutions per genome per day that accumulated in *MUTYH* knockout, *OGG1* knockout, *NUDT1* knockout, and wild-type CHP134 clones. Substitution types are indicated with colors. (*B*) Mutational spectra are displayed according to the 96 substitution types defined by the substitution class and its trinucleotide context for the different knockout and wild-type clones. The substitution types are indicated on the *x*-axis, and the relative contribution of the substitution type is depicted on the *y*-axis.

To validate the correlation between C > A substitutions and defects in the 8-oxoG pathway, we created CRISPR-Cas9 knockout clones of *MUTYH*, *OGG1*, or *NUDT1* in a neuroblastoma cell line (CHP134). To create these knockout clones, we inserted a puromycin resistance cassette in one of the first exons of these genes to disturb the open reading frame (Fig. 5*A*) (21). After puromycin selection, we performed single-cell fluorescence-activated cell sorting (FACS) to create knockout clones (Fig. 5*B*). Knockout clones were genotyped and biallelic editing was confirmed for all selected clones (*SI Appendix*, Fig. S5*A*). The selected clones also showed absent expression of the targeted protein (Fig. 5*C*). Messenger RNA expression of the target gene was also strongly reduced for *MUTYH* and *NUDT1* but only slightly reduced for *OGG1*, possibly because of inefficient nonsense-mediated decay of *OGG1* (*SI Appendix*, Fig. S5 *B–D*). Selected knockout and wild-type clones were passaged for a period between 88 and 126 d to accumulate independent mutations and subsequently single-cell FACS sorted to establish subclones. From both clones and subclones (*n* = 2 per gene), genomic DNA was isolated and subjected to WGS to study the mutational patterns that accumulate between the two clonal steps (*SI Appendix*, Table S3). *MUTYH* and *OGG1* knockout clones showed a relative higher contribution of C > A substitutions ranging from 43 to 57% compared to 25 to 30% in control clones (Fig. 6*A* and *SI Appendix*, Table S3). This difference was statistically significant for the *MUTYH* knockout clones versus the wild-type clones (unpaired *t*-test, two tailed, *P*:0.0076) and the *MUTYH* knockout clones and *OGG1* knockout clones versus the wild-type clones (unpaired *t* test, two-tailed, *P*: 0.0086). Also, the total number of base substitutions per genome per day was increased for *MUTYH* and *OGG1* knockout clones compared to the wild-type clones (*SI Appendix*, Fig. S6*A*). The substitution frequencies in the *NUDT1* knockout clones were similar to the wild-type clones (Fig. 6*A*). No consistent changes in the number of small insertions and deletions were identified between the different knockout and wild-type clones (*SI Appendix*, Fig. S6*B*). These results indicate that knockout of *OGG1* and *MUTYH*, but not *NUDT1*, increases the accumulation of C > A substitutions in individual neuroblastoma cells.

### Mutational Signatures Analysis in Knockout Lines and Neuroblastoma Tumors.

To further study the mutational patterns of the different knockouts, mutational spectra were analyzed and the relative contributions of mutation signatures were determined. The mutational spectra of the wild-type and knockout clones were visualized by showing the relative contribution of the six possible substitutions in their trinucleotide context (Fig. 6*B*). Cosine similarity analysis showed that the mutational spectra of the *MUTYH* and *OGG1* knockouts are slightly different but cluster close together and separately from the wild-type and *NUDT1* knockouts (*SI Appendix*, Fig. S7*A*). The mutational spectra were compared using the COSMIC SigProfiler signatures (6, 22). The *OGG1* knockouts have an increased number of C > A substitutions at the trinucleotide contexts characteristic of signature 18, relative to the wild-type clones. The slightly different pattern of the *MUTYH* knockouts mimics the mutational signature 36 (Fig. 6*B* and *SI Appendix*, Fig. S7*B*).

**Fig. 7.**
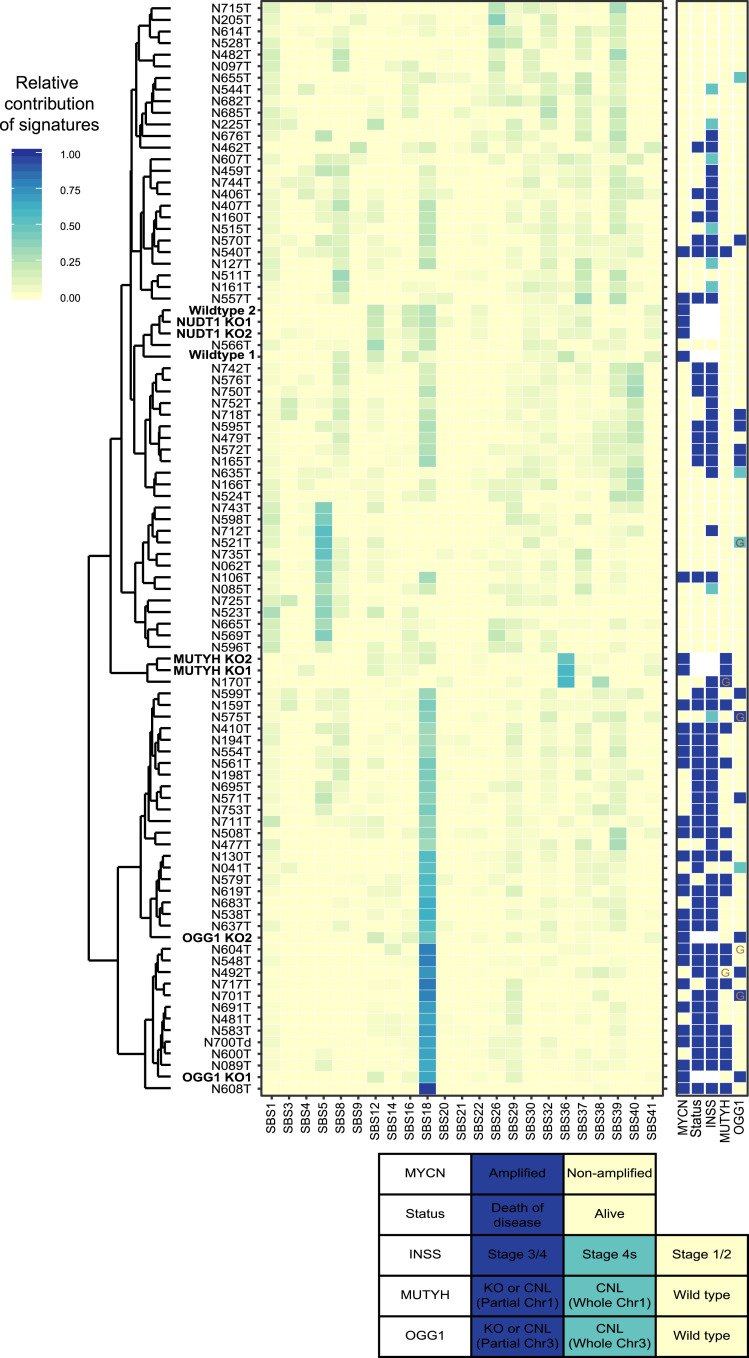
Mutational signatures in neuroblastoma tumors and CHP134 (knockout) clones. Heatmap of neuroblastoma tumors and CHP134 (knockout) clones showing the relative signature contributions for the COSMIC Single Base Substitutions signatures (indicated below the heatmap). Samples have been clustered according to their relative signature contributions. CHP134 clones are indicated in bold. Signature contributions scores are colored as indicated in the color key on the top left. The right panel shows properties of the patient/tumor (*MYCN* status, status [alive or death of disease], INSS stage, *MUTYH* status, and *OGG1* status) represented by color as indicated in the legend below. KO means knockout. A germline mutation in *MUTYH* or *OGG1* is indicated with a G.

To relate the mutational spectra of the knockout clones to primary tumor samples, combined relative signature contributions were determined and plotted in a heatmap (Fig. 7). There is a strong contribution of signature 36 in one of the tumors, which clusters with the *MUTYH* knockout clones (Fig. 7). This tumor (N170T) is the only tumor with both a germline *MUTYH* missense variant and CNL of the other *MUTYH* allele. In addition, a large cluster with a high contribution of signature 18 was identified in our neuroblastoma tumor cohort. These tumors cluster together with the *OGG1* knockout clones (Fig. 7). More than 60% of the tumors in this cluster harbor *OGG1* or *MUTYH* CNL. Furthermore, signature 18 or signature 36 has the highest relative contribution in 90% of the tumors with *OGG1* or *MUTYH* CNL, while this is only 31% in tumors without defective 8-oxoG repair. Overall, these results indicate that the *OGG1* and *MUTYH* knockout clones have a high contribution of C > A mutational signatures 18 and 36, respectively, and cluster together with neuroblastoma tumors with *OGG1* or *MUTYH* CNL.

### Clinical Relevance of Defects in 8-oxoG Repair in Neuroblastoma.

To investigate potential therapeutic strategies for defects in the 8-oxoG repair pathway, we performed a compound screen (∼330 compounds; *SI Appendix*, Table S4 *A* and *B*) with the *OGG1*, *MUTYH*, and *NUDT1* knockout CHP134 clones. No differential sensitivities compared to the CHP134 mother line were identified (*SI Appendix*, Fig. S8 *A* and *B*). These results indicate that defects in *OGG1*, *MUTYH*, and *NUDT1* do not render CHP134 cells more sensitive to chemotherapeutics and targeted compounds. The high frequency of C > A substitutions in neuroblastoma might contribute to the adaptive capacity of the tumors. We evaluated a previously published dataset of primary and relapse neuroblastoma tumors (4). In this cohort of 23 primary-relapse neuroblastoma tumor pairs, 7 of the 15 tumor evolution events in the RAS-MAPK pathway were C > A substitutions (*SI Appendix*, Table S5) (4). This indicates that in this small dataset, 47% of the known single-nucleotide variants (SNVs) involved in tumor evolution are C > A substitutions.

## Discussion

For the majority of pediatric tumors, the most prevalent substitution type is C > T (3). However, C > A substitutions are the most frequent substitution type in neuroblastoma (3, 7). Here, we show that defects in the 8-oxoG repair pathway result in high levels of C > A substitutions in neuroblastoma tumors, which is correlated with a poor prognosis. In concordance, knockout of *OGG1* and *MUTYH* results in increased accumulation of C > A substitutions in a neuroblastoma cell line. We identified a high contribution of C > A mutational signatures 18 and 36 in the *OGG1* and *MUTYH* knockout clones, respectively, which cluster together with neuroblastoma tumors with *OGG1* or *MUTYH* CNL.

CNL of *MUTYH* or *OGG1* is common in neuroblastoma since these genes are located on chromosome arms 1p and 3p, respectively, which are recurrently lost in neuroblastoma (23, 24). On the other hand, *NUDT1* loss (located on 7p) is very rare and, in our cohort, only identified in combination with *MUTYH* CNL. Strikingly, five of the seven tumors with the highest C > A substitution frequencies have multiple alterations in genes of the 8-oxoG repair pathway. This suggests that multiple alterations in this pathway result in a cumulative effect on the C > A substitution frequency. Other neuroblastoma tumors indicate that partial loss of *OGG1* or *MUTYH* can also be sufficient to induce high C > A substitution levels.

We confirmed the effect of defective 8-oxoG repair on the C > A substitution levels in a neuroblastoma cell line. As expected, *MUTYH* and *OGG1* knockout clones showed increased accumulation of C > A substitutions. Most neuroblastoma tumors show heterozygous loss instead of homozygous loss of *OGG1* or *MUTYH*, which we expect to result in a similar but more modest phenotype. The *NUDT1* knockout clones did not show an increase in C > A substitutions compared to the wild-type clones. *NUDT1* prevents the incorporation of 8-oxoG into the DNA. Although 8-oxoG can base pair with both a C and an A, the insertion opposite of an A is expected to be most mutagenic since this will result in A > C (T > G) substitutions, while insertion opposite of a C can be repaired by *OGG1*. Nevertheless, the mutational spectrum of the *NUDT1* knockouts is similar to the wild-type clones, and no increase in T > G substitutions has been detected. This substantiates previous results of *NUDT1* knockout in HAP1 cells, which also did not produce detectable mutational signatures compared to the parental clone (25).

The mutational signature analysis in the knockout clones identified a strong contribution of mutational signatures 18 and 36 in the *OGG1* and *MUTYH* knockout clones, respectively. These similar signatures both mainly consist of C > A substitutions, although with a slightly different trinucleotide distribution (9, 26, 27). Signature 36 was discovered in tumors of patients with *MUTYH*-associated polyposis but has not been described in neuroblastoma tumors (9, 27). A strong contribution of signature 36 was identified in only one of the neuroblastoma tumors, which contains a *MUTYH* missense variant and *MUTYH* CNL. This suggest that only if both *MUTYH* alleles are affected, this results in signature 36, similar to the biallelic variants in patients with *MUTYH*-associated polyposis. Signature 18 was previously identified to be common in neuroblastoma (3, 6, 7, 28, 29). In our cohort of neuroblastoma, signature 18 was also identified as a common signature in a cluster of tumors with frequent CNL of *OGG1* or *MUTYH* (>60% of the tumors). These results confirm the correlation between signatures 18 and 36 and defects in the 8-oxoG repair pathway. In concordance, a *Nudt1*/*Ogg1*/*Mutyh* triple knockout mouse model accumulates C > A substitutions with a mutational pattern that is highly similar to signature 18 or 36 (9, 27, 30). In addition, the study of Brady et al. also identified that tumors with 1p CNL have a significant higher number of substitutions caused by signature 18, while for 3p loss a similar trend was visible (29).

Although in our cohort, most tumors with high C > A substitution frequencies and high contribution of signature 18 have a loss of *OGG1* or *MUTYH*, there are also some tumors with a similar pattern that lack defects in this pathway. This suggests that other factors might play a role. Interestingly, the study of Brady et al. identified enrichment of signature 18 in neuroblastomas with *MYCN* amplification, 17q gain and increased expression of mitochondrial ribosome and electron transport–associated genes (29). Additionally, the level of endogenous oxidative stress might influence whether tumors accumulate high levels of C > A substitutions or not.

The functional consequence of the high frequency of C > A substitutions might lie in the increased adaptive capacity of the tumors. In a previous study, RAS-MAPK alterations, which are enriched in relapsed neuroblastoma, were C > A substitutions in 7 of 15 neuroblastoma tumors with an alteration in this pathway (4, 31). Although this cohort is small, this suggests that C > A substitutions caused by defects in the 8-oxoG repair pathway might influence tumor evolution. Supporting this, Brady et al. showed that in their cohort 52% of driver gene mutations were most likely induced by signature 18, making this the most common cause of driver point mutations in neuroblastoma (29). We propose a tumor evolution model in which early neuroblastoma tumor clones acquire chromosomal aberrations (1p, 3p) that lead to defects in 8-oxoG repair. Subsequently, these tumor cells show an increased frequency of signature 18 and 36 related genomic aberrations that lead to additional defects in tumor evolution associated pathways like RAS-MAPK and ALK.

Although defects in the 8-oxoG repair pathway result in C > A substitutions, no genomic instability is expected, making it unlikely that these defects alter compound sensitivity. As we expected, no differential sensitivities between the *OGG1*, *MUTYH*, or *NUDT1* knockout cells and the CHP134 mother line were identified in our compound screens. We conclude that the C > A substitution phenotype does not function as biomarker for targeted compound intervention to our current knowledge. However, the potential role of this mutational process in tumor evolution in neuroblastoma could select these tumors for ALK or MEK inhibitors (4).

Overall, we identified that defects in the 8-oxoG repair pathway result in high levels of C > A substitutions in neuroblastoma cell line models and tumors. These high levels of C > A substitutions correlate with a poor prognosis, possibly related to an increased adaptive capacity of these tumors.

## Methods

WGS of 86 neuroblastoma tumors was previously performed (17), and percentages of the six possible substitutions were calculated with the actual observations (not corrected for genome distribution). Modified comets assays with neuroblastoma cell lines and organoids were performed as described previously (19). To create knockouts of *MUTYH*, *OGG1*, or *NUDT1* in the CHP134 neuroblastoma cell line, cells were transfected with the guide RNA and Cas9 expressing vector [in pSpCas9n(BB)-2A-GFP] combined with the corresponding homology arm vector (in pJET1.2/blunt vector) to disrupt the open reading frame with a puromycin resistance cassette (21). From clonal and subclonal cultures with confirmed knockout, DNA was isolated and sent for WGS. Mutational landscapes in the mutant clones were explored using an in-house developed R package (MutationalPatterns) (32). To identify mutational signature contributions, the COSMIC SigProfiler signatures (https://www.synapse.org/#!Synapse:syn11967914) were refitted to the obtained mutational profiles for all the clones and tumor samples, and their relative contributions were calculated (excluding low contributing signatures).

*SI Appendix*, *Extended Materials and Methods* includes additional experimental details.

## Supplementary Material

Supplementary File

Supplementary File

## Data Availability

WGS data of the neuroblastoma tumor cohort has been deposited at the European Genome-Phenome Archive (EGA, http://www.ebi.ac.uk/ega/), which is hosted by the EBI, under accession number EGAS00001000222 (33). WGS data of the clones created in this study is available at Sequence Read Archive (https://www.ncbi.nlm.nih.gov/sra/) under accession number PRJNA721261 (34). The raw data of other experiments will be available upon request.
