# Supplementary material for: Defects in 8-oxo-guanine repair pathway cause high frequency of C > A substitutions in neuroblastoma
Source: Proc Natl Acad Sci U S A. 2021 Sep 3;118(36):e2007898118. doi: 10.1073/pnas.2007898118 (PMC8433536; doi:10.1073/pnas.2007898118)
Supplement: Supplementary File [file pnas.2007898118.sapp.pdf]

PNAS
www.pnas.org

Supplementary Information for
Defects in 8-oxo-Guanine repair pathway cause high frequency of C>A substitutions in neuroblastoma

Marlinde L. van den Boogaard[1], Rurika Oka[2], Anne Hakkert[1,3], Linda Schild[1,3], Marli E. Ebus[1,3], Michael R. van Gerven[1], Danny A. Zwijnenburg[3], Piet Molenaar[3], Lieke L. Hoyng[3], M. Emmy M. Dolman[1,3], Anke H.W. Essing[1], Bianca Koopmans[1,3], Thomas Helleday[4,5], Jarno Drost[2], Ruben van Boxtel[2], Rogier Versteeg[3], Jan Koster[3,6], Jan J. Molenaar[1,3,6]

1. Princess Máxima Centre for Paediatric Oncology, Utrecht, Netherlands
2. Oncode Institute, Princess Máxima Center for Pediatric Oncology, Utrecht, the Netherlands
3. Department of Oncogenomics, Academic Medical Center, Amsterdam, the Netherlands.
4. Science for Life Laboratory, Department of Oncology-Pathology, Karolinska Institutet, Stockholm, Sweden
5. Weston Park Cancer Centre, Department of Oncology and Metabolism, University of Sheffield, S10 2RX Sheffield, U.K.
6. These authors contributed equally to this work

To whom correspondence may be addressed. Email:
T.L.vandenBoogaard@prinsesmaximacentrum.nl
J.J.Molenaar@prinsesmaximacentrum.nl

This PDF file includes:

Supplementary text
Figures S1 to S8
Tables S1 to S9
SI References

Supplementary Information Text

Extended materials and methods

Mutation analysis of neuroblastoma tumors

Whole genome sequencing of 86 neuroblastoma tumors was previously performed(1). Percentages of the six possible substitutions were calculated with the actual observations (not corrected for genome distribution). Mutation validations were performed previously with Sanger sequencing(1).

Kaplan scanning for the effect of high or low C>A levels on survival was performed within the R2: Genomics Analysis and Visualization Platform (http://r2.amc.nl) as described previously(1).

To determine whether tumors have a loss of OGG1, MUTYH or NUDT1 a Circular Binary Segmentation (CBS) score with a cutoff of -0.5 was used in the R2 platform.

Generation of *MUTYH* or *OGG1* overexpressing cell lines

Human *OGG1* was cloned from the pENTR1A-OGG1 vector (gift from Helleday lab) into pENTR. The Gateway cloning system was used to insert *OGG1* into the pInducer vector. Human *MUTYH* cDNA was amplified and inserted into pENTR. The Gateway cloning system was used to insert *MUTYH* into the pInducer vector. Mutations in pENTR-OGG1 and pInducer-MUTYH were created with site-directed-mutagenesis and were confirmed with Sanger sequencing. The Gateway cloning system was used to insert mutant OGG1 into the pInducer vector.

For virus production, HEK293T cells were transfected with pInducer-OGG1(wild-type or p.G308E) or pInducer-MUTYH (wild-type or p.A425P) and the packaging plasmids pMD2G, pRRE and pSRV/REV using FuGENE®. DMEM medium containing the virus was collected. Subsequently, SJNB10 and SJNB12 were transduced with the pInducer-MUTYH or pInducer-OGG1 virus, respectively. Blasticidin selection was performed to select for transduced cells, and expression was induced using Doxycycline. Overexpression was confirmed with Western blot.

Modified Comet Assay

For the comet assays neuroblastoma cell lines (SJNB10 and SJNB12) were cultured in DMEM high glucose (41965, ThermoFisher Scientific) supplemented with 10% FBS (10500, Gibco by Life Technologies), 100 U/mL Penicillin with 100 mg/mL Streptomycin (15140, Gibco by Life Technologies), 1x NEAA (11140, Gibco,Thermo Fischer Scientific) and 2 mM L-glutamine (25030, Gibco by Life Technologies). Tumor-derived-organoids AMC691T, AMC700T and AMC717T were cultured in DMEM (21885, Gibco by Life Technologies) with 20% Ham's F-12 Nutrient Mix (31765, Gibco by Life Technologies), supplemented with 1x B-27^{TM} Supplement (12587, Gibco by Life Technologies), 100 U/mL Penicillin with 100 mg/mL Streptomycin (15140, Gibco by Life Technologies), 20 ng/mL Animal-Free Recombinant Human EGF (AF-100-15, Peprotech), and 40 ng/mL Recombinant Human FGF-basic (100-18B, Peprotech).

The modified comets assays with neuroblastoma cell lines and organoids were performed as described previously(2). During the assay, slides were incubated with buffer alone or with recombinant OGG1, which was supplied by the Helleday lab (figures 4A,B,C, supplementary figure 4A) or from Abcam (ab98249, supplementary figure 4B) (2). As a counterstain 60 µl of Sybr^{TM} Gold Nucleic Acid Gel Stain (S11494, Thermo Scientific, diluted 1:1000 in PBS) was used. The percentage of DNA in the tail and the tail moment is quantified using the Comet Score^{TM} software (TriTek).

Vector construction for CRISPR cas9 editing

The pSpCas9n(BB)-2A-GFP vector was obtained from Addgene (#48138). gRNA sequences are listed in table S6. For insertion of the gRNA, the vector was digested with BbsI (New England Biolabs). gRNA oligo's were annealed in a hybridization reaction and were ligated into the vector after digestion of the vector with BbsI (New England Biolabs).

Homology arms and the puromycin cassette were cloned into the pJET1.2/blunt vector (Thermo Scientific) as described previously(3). The primers used for amplification of the homology arms are listed in table S7. PCR reactions were performed using the Phusion High-Fidelity DNA Polymerase (Thermo Scientific). The 3' homology arm product was ligated directly into the blunt

ends of the Pjet1.2/blunt vector. Next, this vector was digested with NotI (GQ, R643E, Promega) and the 5' homology arm PCR product was cloned into the multiple cloning site using the Infusion HD cloning kit (Takara Bio). Subsequently, the vector was digested with XhoI (R616A, Promega) and the PGK-EGFP-Puromycin cassette was cloned into the multiple cloning site using Infusion HD cloning.

CHP134 transfection and clonal cultures

The neuroblastoma cell line CHP134 was cultured in DMEM high glucose (41965, ThermoFisher Scientific) supplemented with 10% FBS (10500, Gibco by Life Technologies), 100 U/mL Penicillin with 100 mg/mL Streptomycin (15140, Gibco by Life Technologies), 1x NEAA (11140, Gibco,Thermo Fischer Scientific) and 2 mM L-glutamine (25030, Gibco by Life Technologies). CHP134 was transfected with the gRNA and Cas9 expressing vector (in pSpCas9n(BB)-2A-GFP) combined with the corresponding homology arm vector (in pJET1.2/blunt vector) using Fugene HD transfection reagent (E2312, Promega). Three days after transfection medium containing 0.25 µg/ml Puromycin (Sigma; Cat#: P8833) was added to the cells. Eleven days after transfection the cells were single cell sorted by Flow cytometry with the Cell Sorter SH800S (SONY). Clonal cultures were expanded and harvested for DNA, RNA and protein to confirm genome editing. Clones with biallelic editing were subjected to a second round of single cell sorting between 88 and 126 days after the first sort to expand subclones. From both clonal and subclonal cultures cells were harvested for genomic DNA isolation for whole genome sequencing.

Genotyping of clones

For genotyping, genomic DNA was isolated using the Wizard® SV Genomic DNA Purification System (Promega). Primers were designed (table S8) to amplify the allele with PGK-EGFP-Puromycin insertion or the allele with wild-type size. PCR products were analyzed by gel electrophoresis and Sanger sequencing.

Western blot analysis

Samples were harvested in Laemmli buffer and subjected to Western blotting. Samples were run on 10% TGX Stain-Free™ Precast Gels (Bio-rad). Proteins were transferred on PVDF membranes (Trans-Blot® Turbo™ RTA Transfer Kit (Bio-Rad; Cat#: 170-4272)) using the Trans Blot Turbo Transfer System (Bio-rad). Membranes were blocked using ECL advance blocking reagent (RPN418V, GE Healthcare) in TBS-Tween 0.1%. The following primary antibodies were used: anti-NUDT1 (anti-MTH1, NB100-109, Novus Biologicals), anti-OGG1 (ab124741, Abcam), anti-MUTYH (anti-MYH, 4D10, Novus Biologicals), anti-α-Tubulin (3873S, Cell signaling), anti-GAPDH (14C10, Cell Signaling) and anti-β-actin (Ab6276, Abcam). The following secondary antibodies were used: Mouse IgG HRP Linked Whole Ab (NXA931-V, GE Healthcare), Rabbit IgG HRP linked F(ab)'2 (NA9340-V, GE Healthcare), IRDye® 680RD Goat Anti Rabbit (926-68071, LI-COR) and IRDye® 680RD Goat Anti Mouse (926-68070, LI-COR). Western blots were imaged using the Bio-Rad Chemidoc™ Touch (Bio-Rad) with the ECL-detection kit (GE Healthcare) and/or the Odyssey CLx (LI-COR). The western blots were analyzed using the Image Lab Software (Bio-Rad) and Image Studio Software (LI-COR). Loading controls were visualized on the same membranes as the proteins of interest.

qRT-PCR

Cells were harvested in Trizol (Ambion by Life Technologies) and RNA was isolated with a standard chloroform extraction. One microgram RNA was used for cDNA synthesis using the IScript cDNA Synthesis Kit (1708891, Bio-Rad) according to the manufacturer's protocol. qRT-PCRs were performed on a C1000 thermal cycler (Bio-Rad) using SYBR green (1708886, Bio-Rad). Data was analyzed using the CFX Maestro Software (Bio-Rad). Expression levels were normalized to *HPRT1* and *UBC* expression. Primer sequences can be found in Table S9.

DNA isolation and whole genome sequencing of cell line

For DNA isolation of clones and subclones, cells were harvested in 1x SE buffer (75mM NaCl; 25mM Na2 EDTA; pH 8.0) and DNA was isolated using a standard chloroform extraction protocol. Subsequently, an extra DNA purification was performed using the DNA blood and tissue kit

(69504, Qiagen) including an RNAseA incubation (19101, Qiagen). Clones and subclones were sequenced (2x 150 bp) with the Illumina Xten at the Hartwig Medical Foundation.

Read alignment, variant calling and variant filtering

Sequence reads were mapped against human reference genome GRCh37 by using Burrows-Wheeler Aligner v0.5.9 mapping tool(4) with settings 'bwa mem -c 100 -M'. Sequence reads were marked for duplicates by using Sambamba v0.4.732 and realigned per donor by using Genome Analysis Toolkit (GATK) IndelRealigner v2.7.2. Raw variants were multisample-called by using the GATK HaplotypeCaller v3.4-46(5) and GATK-Queue v3.4-46 with default settings and additional option 'EMIT_ALL_CONFIDENT_SITES'. The quality of variant and reference positions was evaluated by using GATK VariantFiltration v3.4-46 with options '-snpFilterName LowQualityDepth -snpFilterExpression "QD < 2.0" -snpFilterName MappingQuality -snpFilterExpression "MQ < 40.0" -snpFilterName StrandBias -snpFilterExpression "FS > 60.0" -snpFilterName HaplotypeScoreHigh -snpFilterExpression "HaplotypeScore > 13.0" -snpFilterName MQRankSumLow -snpFilterExpression "MQRankSum < −12.5" -snpFilterName ReadPosRankSumLow -snpFilterExpression "ReadPosRankSum < −8.0" -cluster 3 -window 35'. Full pipeline description and settings also available at: https://github.com/UMCUGenetics/IAP.

To obtain high-quality somatic mutation catalogs, we applied postprocessing filters as described(6). Briefly, we considered variants at autosomal chromosomes without any evidence from a paired control sample (i.e., clone from which the subclone is derived); passed by VariantFiltration with a GATK phred-scaled quality score ≥50 for base substitutions and ≥250 for indels; a base coverage of at least 20X in the sub-clonal and paired control sample; mapping quality (MQ) of ≥60; no overlap with single nucleotide polymorphisms (SNPs) in the Single Nucleotide Polymorphism Database v137.b3730; and absence of the variant in a panel of unmatched normal human genomes (BED-file available upon request). We additionally filtered base substitutions with a GATK genotype score (GQ) lower than 99 or 10 in sub-clonal or paired control sample, respectively. For indels, we filtered variants with a GQ score lower than 99 in both sub-clonal and paired control sample and filtered indels that were present within 100 bp of a called variant in the control sample. In addition, for both

SNVs and INDELs, we only considered variants with a variant allele frequency of 0.3 or higher in the sub-clones to exclude mutations that were already present in the parental clone (6, 7).

Mutational profile and signature analysis

Mutational landscapes in the mutant clones were explored using an in-house developed R package (MutationalPatterns)(8), such as mutation load, distribution throughout the genome, mutation types, transcription strand biases and replication timing and strand biases. The consensus replication map included in the package was used to quickly check for any significant replication strand bias.

To identify mutational signature contributions, the COSMIC SigProfiler signatures (https://www.synapse.org/#!Synapse:syn11967914) were refitted to the obtained mutational profiles for all the clones and tumor samples and their contributions were calculated. Then, signatures contributing at least 100 variants in a sample were selected and refitted again to exclude low contributing signatures. The relative contributions of the selected signatures were plotted as a heatmap. To determine similarity in mutational patterns between samples, the cosine similarity against each other was calculated.

Compound screen

For the compound screen equal amounts of cells of four CHP134 clones with knockout of the same gene were combined. As a control the CHP134 mother line was used. Cells were seeded in 384 well plates at a density of 2000 cells/well. The next day library compounds (Tables S4A,B) were added to obtain a final concentration between 0.1 nm to 10 mM using the Sciclone 3000 liquid handling robot. Control samples were treated with appropriate concentrations of solvents. Cells were incubated with compounds for 72 hours. Cell viability was measured before and after 72 hours of compound treatment with a 3-(4.5-dimethylthiazol-2-yl)-2,5-diphenyltetrazolium bromide (MTT) colorimetric assay. Area under the curve (AUC) values were calculated using Graphpad Prism.

Statistics section

The logrank test was used to determine the p-value for the difference in overall survival between patients with high or low C>A frequency tumors. The unpaired T-test with Welch correction (two-sided) was used to determine p-values for C>A frequency vs stage, for C>A frequency vs MUTYH/OGG1 status and for the comet assays. The unpaired T-test (two-sided) was used to determine p-values for C>A frequency of the wild-type vs knockout clones. Statistical tests were performed in Graphpad Prism.

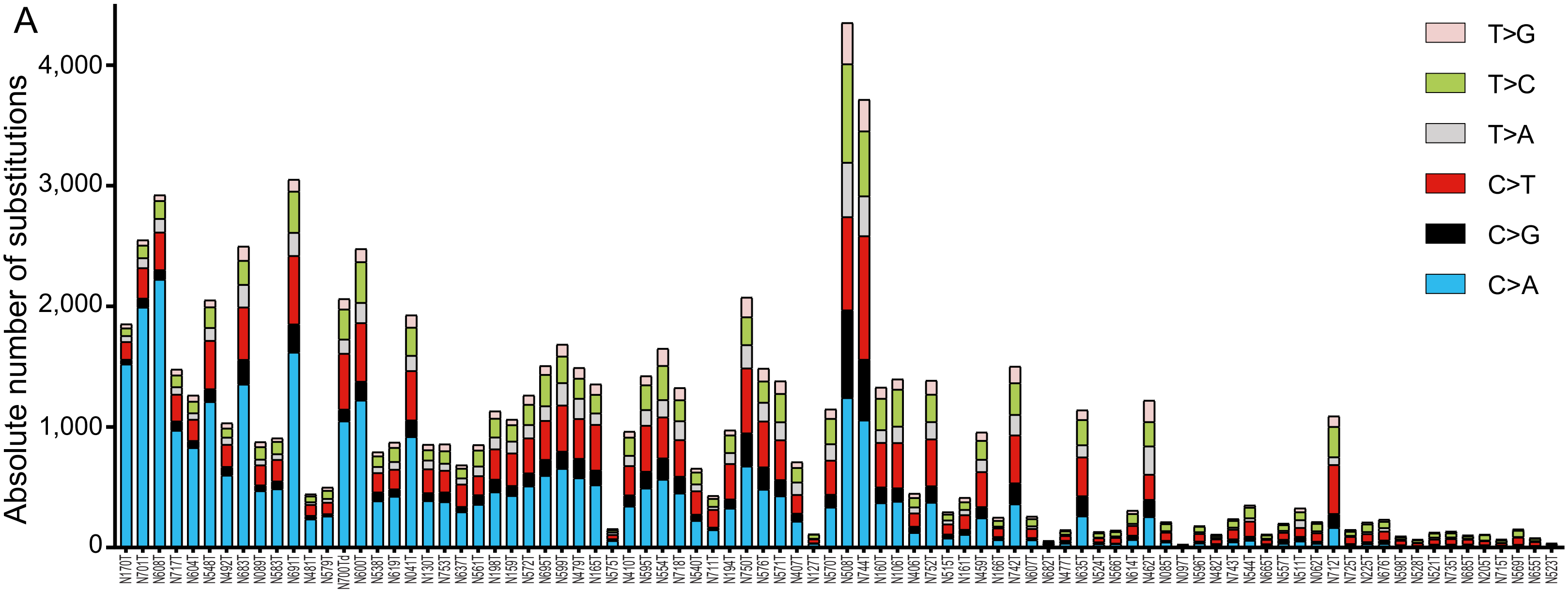

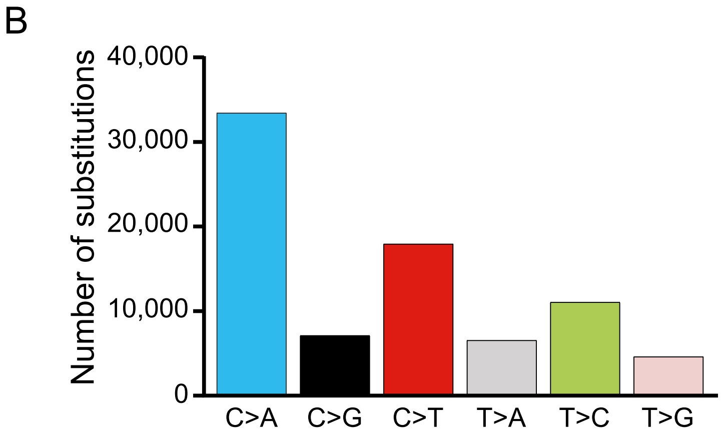

C

	Validated	Total	Ratio
A>C	9	11	82%
A>G	23	29	79%
A>T	18	19	95%
C>A	148	176	84%
C>G	30	32	94%
C>T	87	96	91%

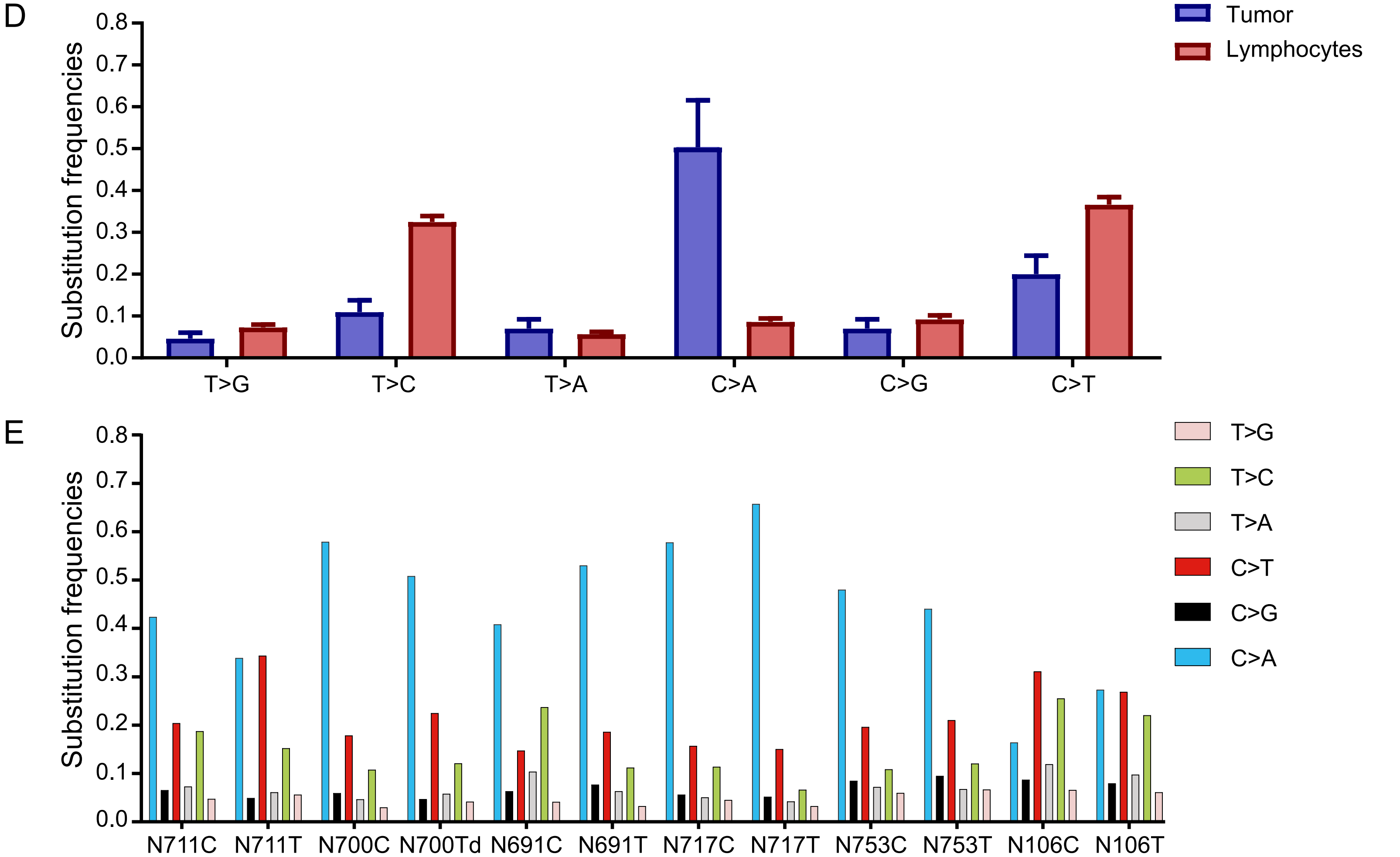

Fig. S1. Confirmation of high frequency of C>A substitutions in neuroblastoma tumors. *(A)* Absolute number of of the six possible nucleotide substitutions, indicated with different colors, in a cohort of 86 neuroblastoma tumors. Tumors are ranked from left to right on C>A substitution frequency as in figure 1A. *(B)* Total number of the six different substitutions identified in 86 neuroblastoma tumors. *(C)* Confirmation of nucleotide substitutions by Sanger sequencing, shown are the number of substitutions validated by Sanger sequencing (Validated) and the total number substitutions tested by Sanger sequencing (Total) and the ratio between validated/total (Ratio). *(D)* Average substitution frequencies of the different nucleotide substitutions in tumor DNA corrected for lymphocyte DNA (blue bars) and in lymphocytes corrected for tumor DNA (red bars) for 26 tumors with a high C>A frequency in tumor DNA corrected for lymphocyte DNA. *(E)* Comparison of substitution frequency for tumor (T) and cell line pairs (C) indicates that cell lines have a similar C>A substitution frequency as their corresponding tumor. Substitution types are indicated by color.

A

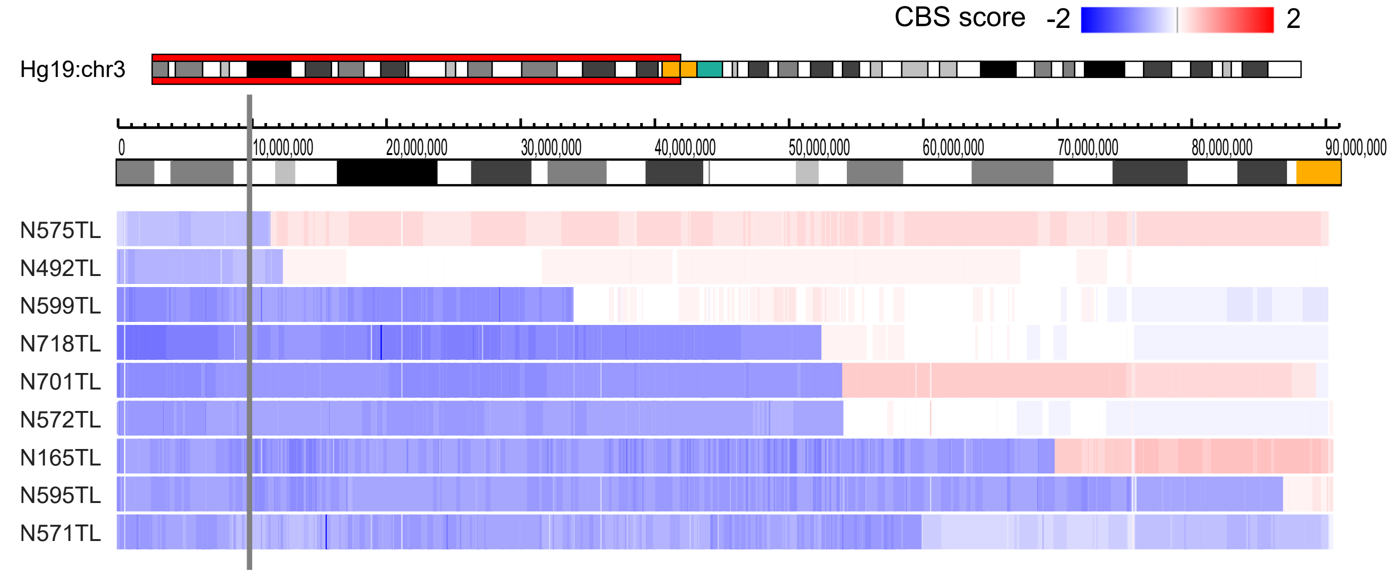
CBS score -2 2
Hg19:chr3
0
10,000,000
20,000,000
30,000,000
40,000,000
50,000,000
60,000,000
70,000,000
80,000,000
90,000,000
N575TL
N492TL
N599TL
N718TL
N701TL
N572TL
N165TL
N595TL
N571TL

B

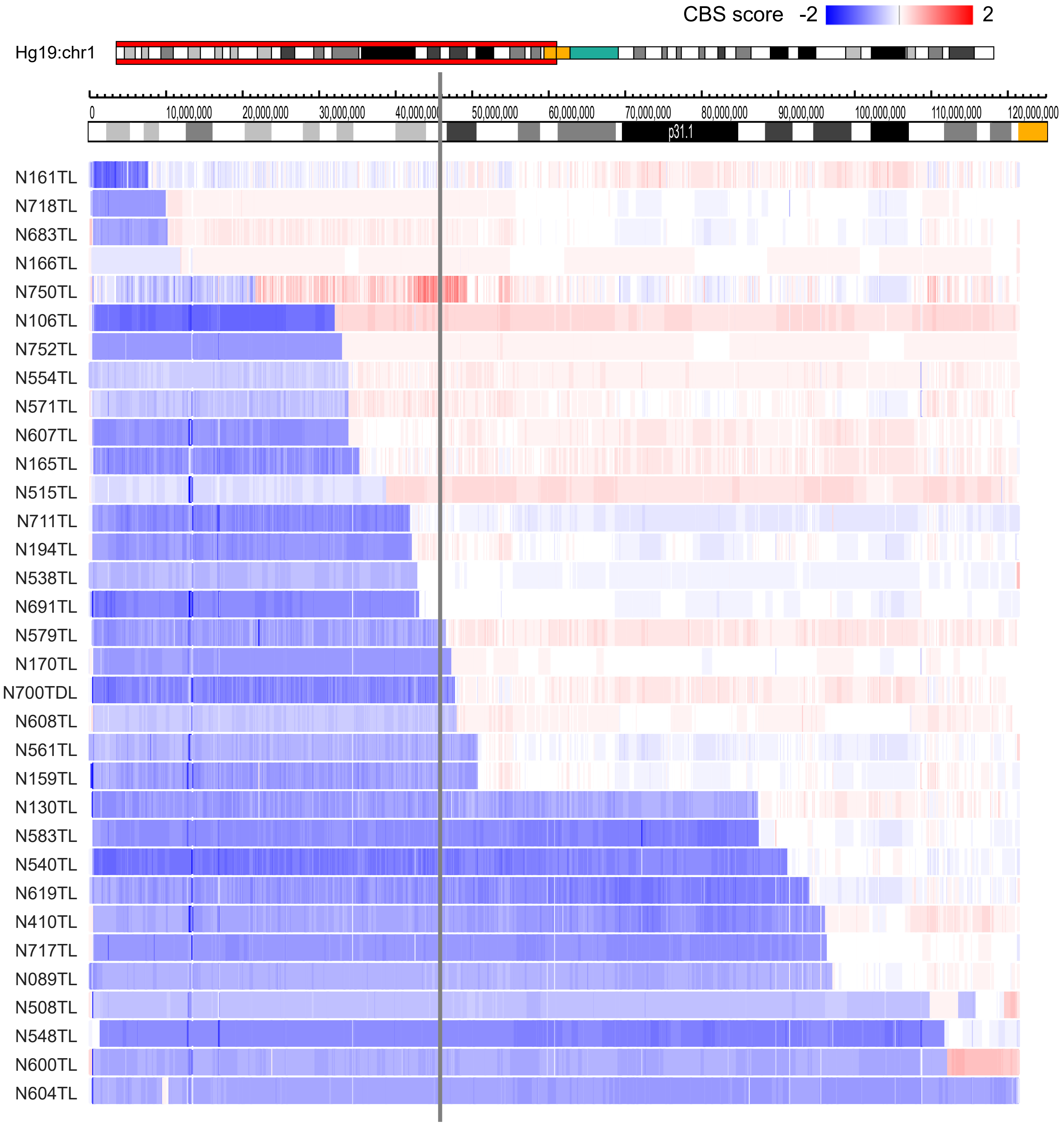
CBS score -2 2
Hg19:chr1
0
10,000,000
20,000,000
30,000,000
40,000,000
50,000,000
60,000,000
70,000,000
80,000,000
90,000,000
100,000,000
110,000,000
120,000,000
p31.1
N161TL
N718TL
N683TL
N166TL
N750TL
N106TL
N752TL
N554TL
N571TL
N607TL
N165TL
N515TL
N711TL
N194TL
N538TL
N691TL
N579TL
N170TL
N700TDL
N608TL
N561TL
N159TL
N130TL
N583TL
N540TL
N619TL
N410TL
N717TL
N089TL
N508TL
N548TL
N600TL
N604TL

Fig. S2. Overview of 3p and 1p loss in neuroblastoma tumors. *(A)* Overview of tumors with 3p loss in the cohort of neuroblastoma tumors used in this study. The grey line indicates the position of *OGG1*. Blue indicates a Circular Binary Segmentation (CBS) score <0 (deletion) and red indicates a CBS score >0 (gain), as indicated by the color scale. *(B)* Overview of tumors with 1p loss in the cohort of neuroblastoma tumors used in this study. The grey line indicates the position of *MUTYH*. Colors same as in A.

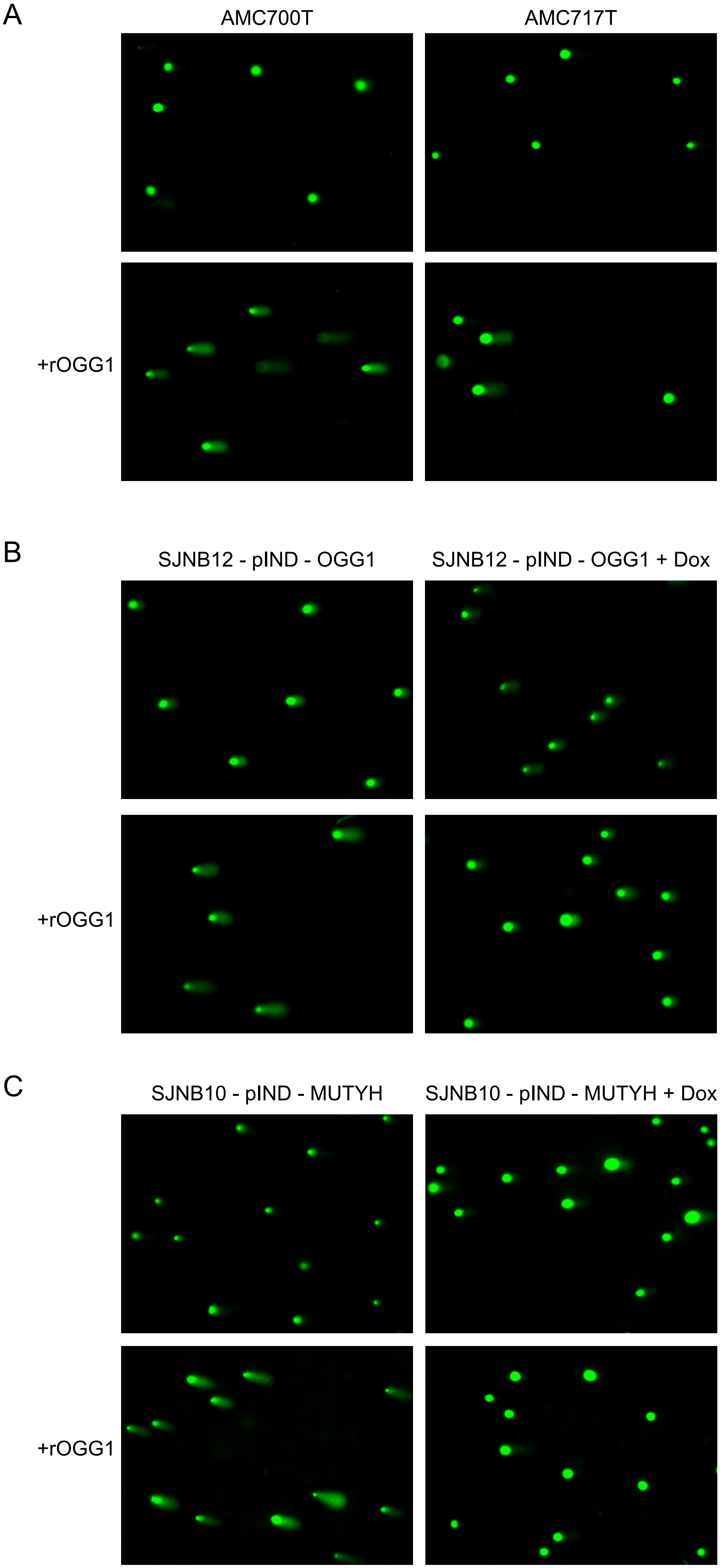
A
AMC700T
AMC717T
AMC691T
+rOGG1
B
SJNB12 - pIND - OGG1
SJNB12 - pIND - OGG1 + Dox
+rOGG1
C
SJNB10 - pIND - MUTYH
SJNB10 - pIND - MUTYH + Dox
+rOGG1

Fig. S3. Defects in the 8-oxoG repair pathway results in higher 8-oxoG levels. *(A)* Representative pictures of modified comet assays (with and without recombinant OGG1 (rOGG1)) with two organoids with defects in the 8-oxoG pathway (AMC717T with MUTYH copy number loss, AMC700T with OGG1 copy number loss) and one organoid with a functional 8-oxoG pathway (AMC691T). *(B)* Representative pictures of modified comet assays (with and without recombinant OGG1 (rOGG1)) of SJNB12 with or without Dox induced OGG1 overexpression. *(C)* Representative pictures of modified comet assays (with and without recombinant OGG1 (rOGG1)) of SJNB10 with or without Dox induced MUTYH overexpression.

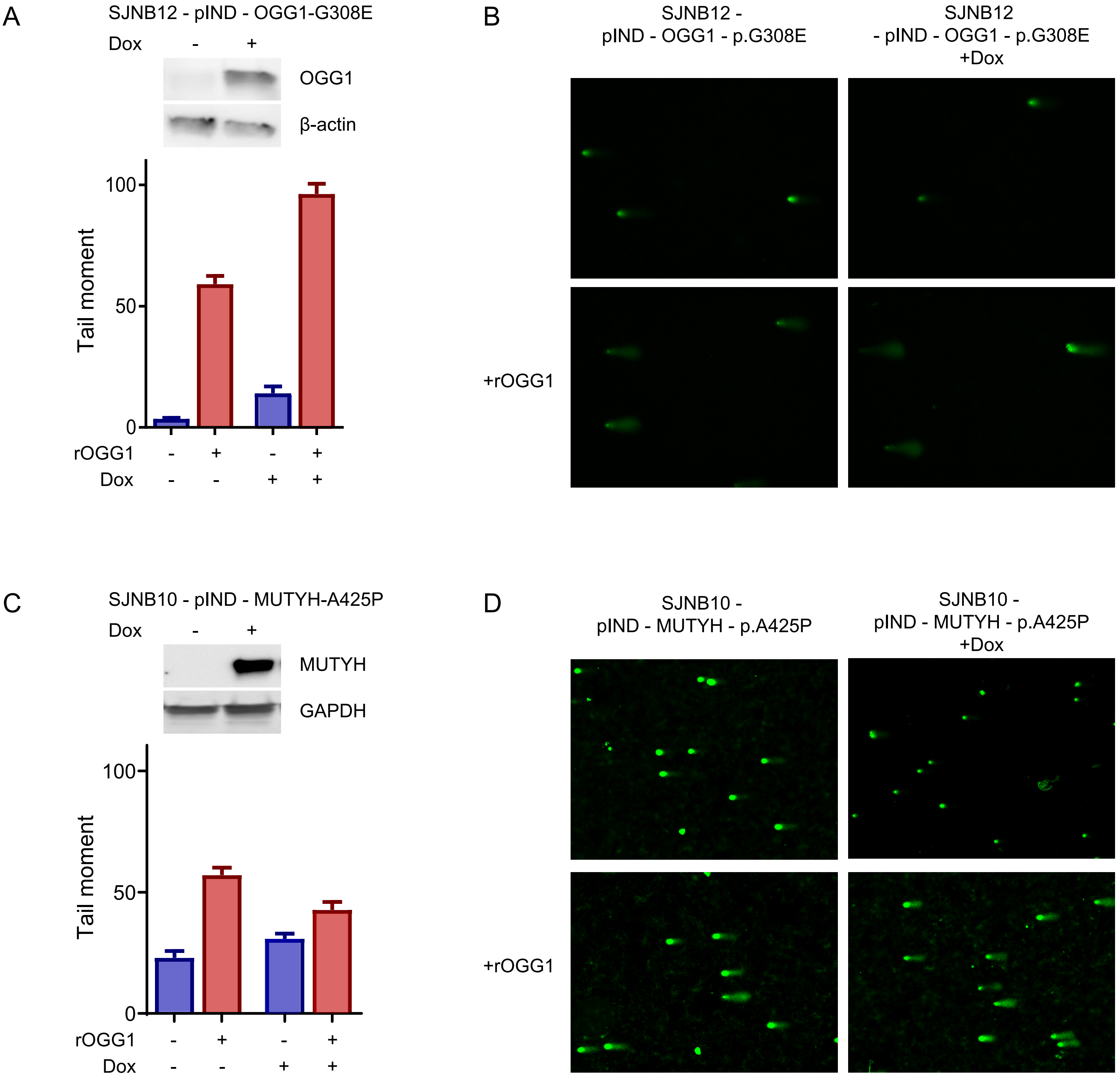

Fig. S4. Mutant OGG1 and mutant MUTYH do not completely rescue high 8-Oxo-Guanine levels in SJNB12 and SJNB10 respectively. *(A)* Tail moment calculated from modified comet assays with (red bar (+)) or without (blue bar (-)) recombinant OGG1 (rOGG1) treatment. Upper panel shows mutant OGG1 (p.G308E) overexpression in SJNB12 transduced with a pIND-OGG1- G308E vector. β-actin is used as a loading control. Lower panel shows tail moments calculated from modified comet assays for SJNB12 with (+) or without (-) Dox induced OGG1-G308E overexpression. *(B)* Representative pictures of comet assays described in panel A. *(C)* Tail moment calculated from modified comet assays with (red bar (+)) or without (blue bar (-)) recombinant OGG1 (rOGG1) treatment. Upper panel shows mutant MUTYH (p.A425P) overexpression in SJNB10 transduced with a pIND-MUTYH- A425P vector. GAPDH is used as a loading control. Lower panel shows tail moments calculated from modified comet assays for SJNB12 with (+) or without (-) Dox induced MUTYH-A425P overexpression. *(D)* Representative pictures of comet assays described in panel C. Error bars indicate the standard error of the mean.

A

Clone	gRNA	Allele 1	Allele 2
NUDT1 KO1	NUDT1 gRNA2	Puromycin	Deletion 8 nt
NUDT1 KO2	NUDT1 gRNA1	Puromycin	Puromycin
OGG1 KO1	OGG1 gRNA2	Puromycin	Deletion 16 nt
OGG1 KO2	OGG1 gRNA1	Puromycin	Deletion 2 nt
MUTYH KO1	MUTYH gRNA3	Puromycin	Puromycin
MUTYH KO2	MUTYH gRNA4	Puromycin	Puromycin

B

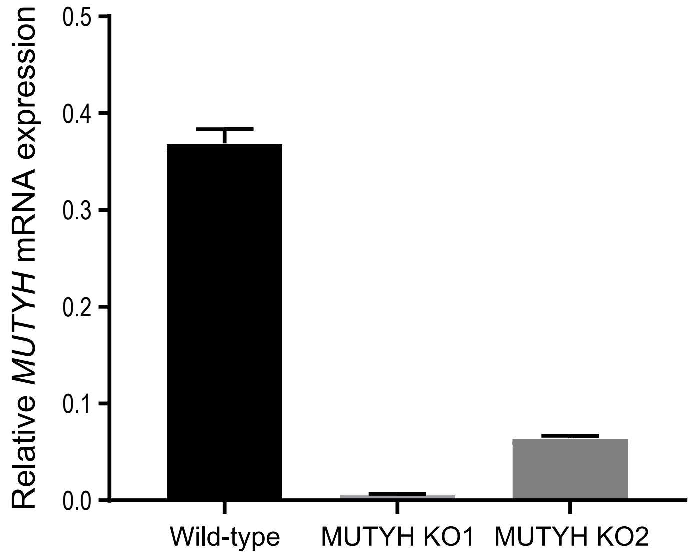

C

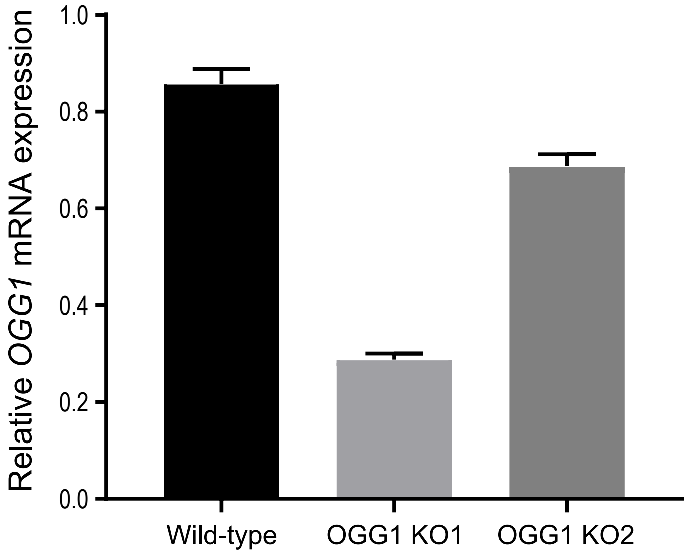

D

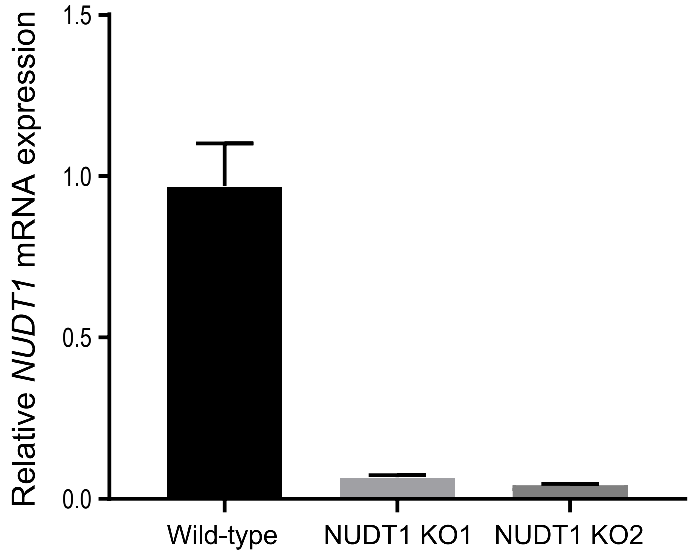

Fig. S5. Genome editing and RNA expression in CHP134 clones. *(A)* Genotyping results of the single cell clones with the indicated gRNA. PCR followed by Sanger sequencing identified puromycin insertions and small deletions in the selected clones. *(B) MUTYH* mRNA expression levels measured with q-PCR for wild-type and *MUTYH* knockout clones. Expression levels are normalized to HPRT1 and UBC. *(C)* Same as in B, but for *OGG1*. *(D)* Same as in B, but for *NUDT1*. Error bars represent standard error of the means of three technical replicates.

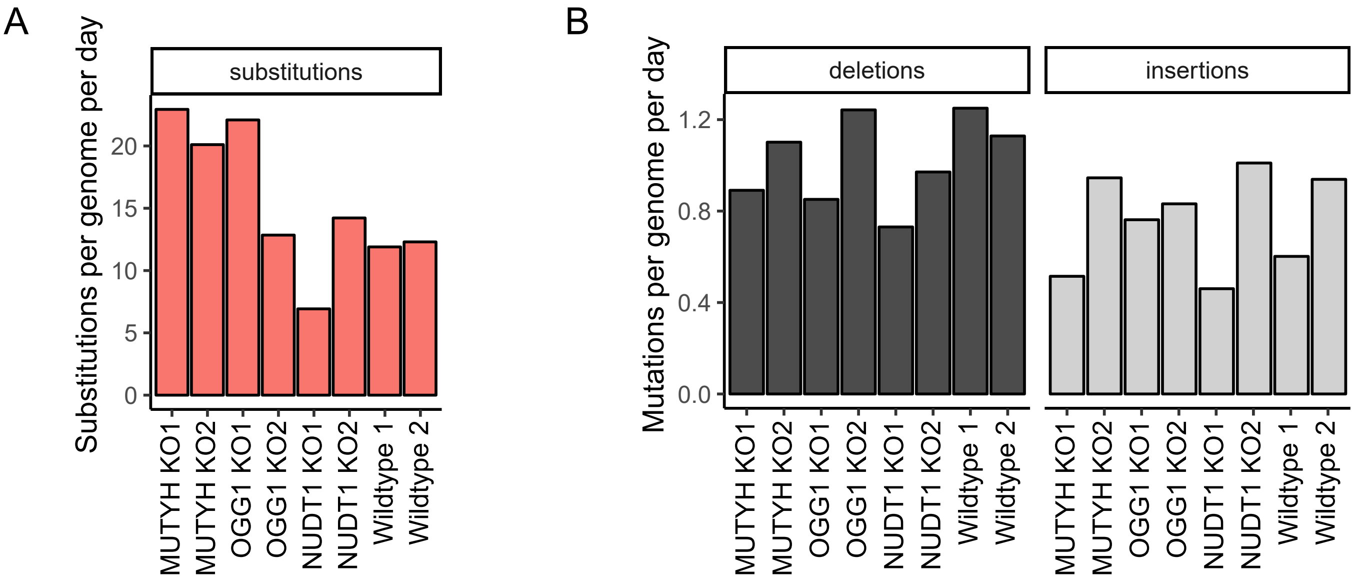

Fig. S6. Substitutions, insertions and deletions in CHP134 (knockout) clones. *(A)* Total number of base substitutions per genome per day that accumulated in *MUTYH* knockout, *OGG1* knockout, *NUDT1* knockout and wild-type CHP134 clones. *(B)* Total number of insertions and deletions (1 bp insertion/deletion up to 150 bp deletion and 192 bp insertion) per genome per day that accumulated in *MUTYH* knockout, *OGG1* knockout, *NUDT1* knockout and wild-type CHP134 clones.

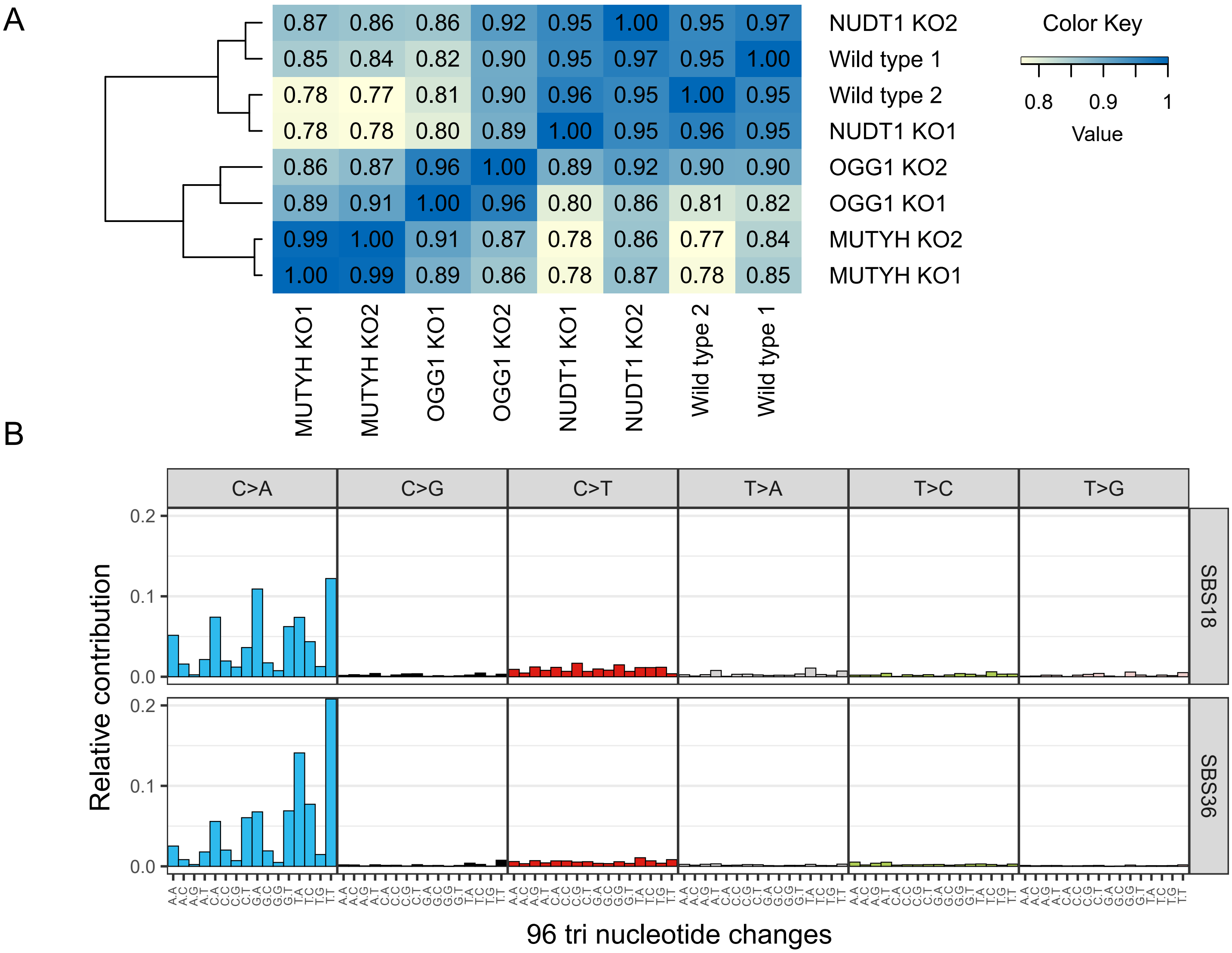

Fig. S7. Similarity of mutational spectra in CHP134 clones. *(A)* Heatmap showing the cosine similarity scores for the mutational patterns of the different CHP134 (knockout) clones. Heatmap is colored as indicated in the color key. *(B)* Mutational spectra of mutational signatures 18 and 36 (9,10). Mutational spectra are displayed according to the 96 substitution types defined by the substitution class and its trinucleotide context for the different knockout and wild-type clones. The substitution types are indicated on the x-axis and the relative contribution of the substitution type is depicted on the y-axis.

A

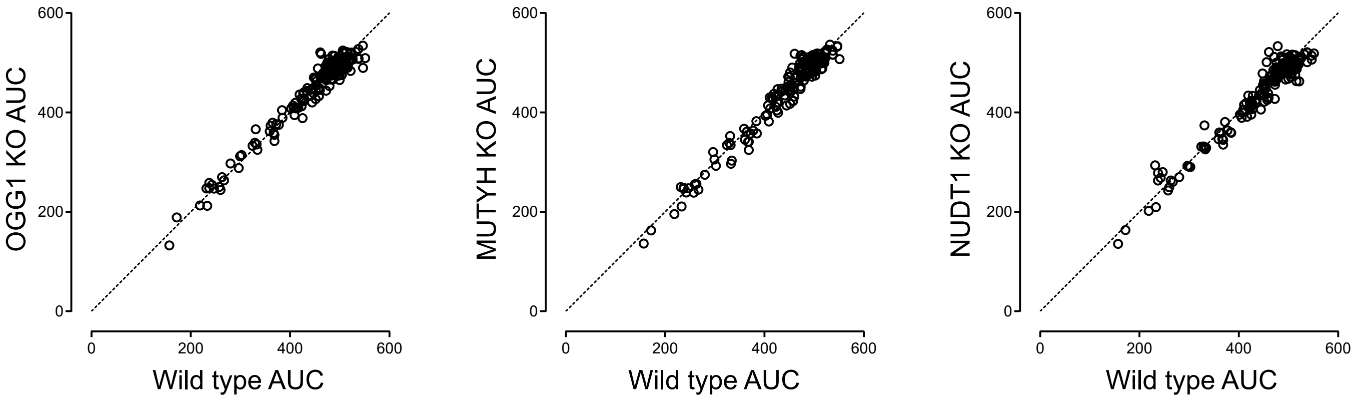

B

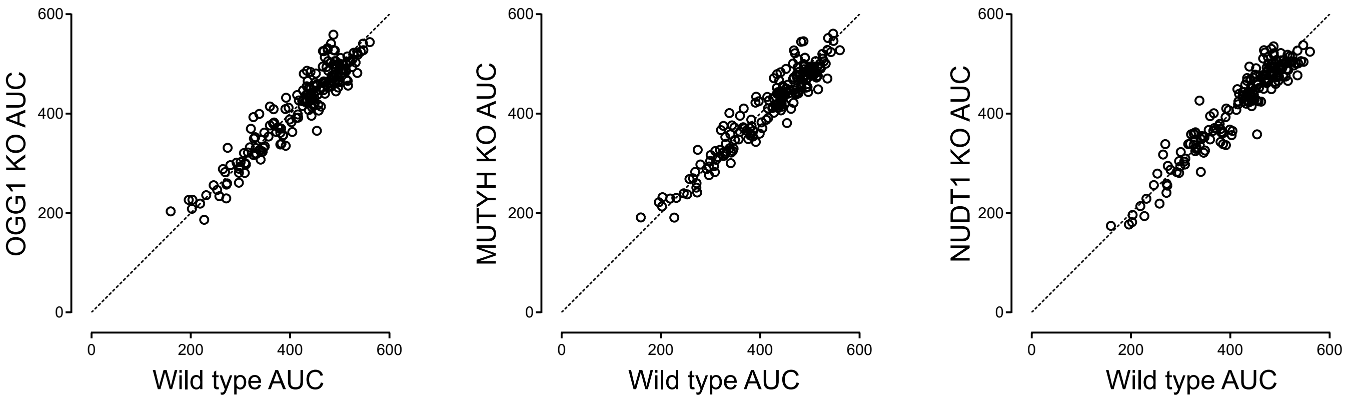

Fig. S8. Compound sensitivities of knockout compared to wild-type CHP134. *(A)* Area under the curve (AUC) values of a standard compound library (Table S4A) plotted for *OGG1* knockout vs wild-type CHP134 (left), *MUTYH* knockout vs wild-type CHP134 (middle) and *NUDT1* knockout vs wild-type CHP134 (right). AUC values are the average of two technical replicates. For the compound screen four different knockout clones per gene were pooled before plating. *(B)* Same as in (A) for DNA damage compound library (Table S4B).

Table S1: Cox regression for survival analysis. Age group = <18 months vs >18 months, C>A = low vs high, inss = st1,2,3,4s vs 4.

**variable 1**			**variable 2**		
variable 1	Hazard ratio (95% CI)	p-value	variable 2	Hazard ratio (95% CI)	p-value
**univariate**					
MYCN amp	6.546 (3.317-12.92)	6.04E-08			
C>A	14.89 (5.707-38.84)	3.40E-08			
Age group	13.36 (4.634-38.51)	1.60E-06			
inss	17.17 (5.17-57.03)	3.44E-06			
**multivariate**					
C>A	10.566 (3.752-29.759)	8.09E-06	MYCN amp	2.088 (1.022-4.267)	4.35E-02
C>A	7.181 (2.537-20.32)	2.04E-04	Age group	5.159 (1.623-16.40)	5.43E-03
C>A	6.103 (2.101-17.73)	8.87E-04	inss	5.989 (1.558-23.02)	9.17E-03

Table S2: C>A substitution frequencies and CBS scores for MUTYH, OGG1 and NUDT1 for neuroblastoma tumors.

		MUTYH			OGG1			NUDT1			
Tumor	C>A substitution frequency	Status	Partial / whole chromosome	CBS score	Status	Partial / whole chromosome	CBS score	Status	Partial / whole chromosome	CBS score	Germline events
N170TL	0.8202	Deletion	Partial	-0.8398			0.0391			0.0486	MUTYH missense variant (NP_036354.1:p.Ala425Pro)
N701TL	0.7810			0.0307	Deletion	Partial	-0.8567			0.7943	OGG1 missense variant (NP_002533.1:p.Gly308Glu)
N608TL	0.7604	Deletion	Partial	-0.5475			-0.1264			0.2036	
N717TL	0.6572	Deletion	Partial	-0.8381			0.018	Deletion	Partial	-0.8526	
N604TL	0.6550	Deletion	Partial	-0.7439			0.0317			0.1014	OGG1 missense variant (NP_002533.1:p.Arg46Gln)
N548TL	0.5893	Deletion	Partial	-0.9678			-0.0644			-0.0621	
N492TL	0.5790			0.0442	Deletion	Partial	-0.5971			0.5091	MUTYH missense variant (NP_036354.1:p.Tyr176Cys)
N683TL	0.5417			0.0605			0.099			0.146	
N089TL	0.5349	Deletion	Partial	-0.6579			-0.0092			0.0668	
N583TL	0.5342	Deletion	Partial	-0.9329			0.0423			0.0883	
N691TL	0.5302			-0.0979			-0.1058			-0.1605	
N481TL	0.5294			-0.12			0.0507			0.3439	
N579TL	0.5181	Deletion	Partial	-0.8543			-0.0781	Deletion	Partial	-0.8216	
N700TDL	0.5080	Deletion	Partial	-1.1225			-0.2092			-0.2434	
N600TL	0.4927	Deletion	Partial	-0.6549			-0.1927			0.41	
N538TL	0.4867			-0.0286			-0.1252			-0.0535	
N619TL	0.4856	Deletion	Partial	-0.773			0.1047			0.2038	
N041TL	0.4761			-0.0339	Deletion	Whole	-0.5746			0.4511	
N130TL	0.4525	Deletion	Partial	-0.8789			-0.1017			-0.1036	
N753TL	0.4404			0.0313			0.0927			0.1675	
N637TL	0.4296			-0.1538			-0.1544			0.4075	
N561TL	0.4188	Deletion	Partial	-0.5411			0.1162			0.1733	
N198TL	0.4062			-0.1503			-0.0959			0.8302	
N159TL	0.4034	Deletion	Partial	-0.7029			0.1132			0.2172	
N572TL	0.4021			0.0306	Deletion	Partial	-0.7106			0.5072	
N695TL	0.3951			-0.1122			-0.1153			0.1626	
N599TL	0.3880			0.03	Deletion	Partial	-0.724			0.0971	
N479TL	0.3859			-0.2894			-0.2797			-0.4516	
N165TL	0.3821			0.0154	Deletion	Partial	-0.9838			0.4692	
N575TL	0.3571			-0.1419	Deletion	Partial	-0.6381			0.1508	OGG1 missense variant (NP_002533.1 p.Val267Met)

N410TL	0.3548	Deletion	Partial	-0.7573			-0.159			0.2975	
N595TL	0.3448			-0.4026	Deletion	Partial	-0.8784			0.2012	
N554TL	0.3420			0.0488			-0.472			0.0332	
N718TL	0.3394			0.0494	Deletion	Partial	-0.8745			0.2017	
N540TL	0.3389	Deletion	Partial	-1.1519			-0.2448			-0.2607	
N711TL	0.3388			0.018			-0.0101			0.0955	
N194TL	0.3333			0.0709			0.1297			0.1866	
N750TL	0.3247			0.9236			-0.4306			0.5196	
N576TL	0.3243			0.0194			-0.044			0.2397	
N571TL	0.3089			0.2416	Deletion	Partial	-0.5516			0.5836	
N407TL	0.3051			0.0264			-0.044			-0.0699	
N127TL	0.2909			-0.3688			-0.3492			0.5043	
N570TL	0.2906			-0.1809	Deletion	Whole	-0.5113			0.1007	
N508TL	0.2853	Deletion	Partial	-0.5192			-0.314			0.044	
N744TL	0.2841			0.4755			-0.4281			0.5031	
N160TL	0.2781			0.0734			0.0841			0.5074	
N106TL	0.2731			0.2742			-0.2347			0.7347	
N406TL	0.2729			-0.0051			-0.2654			0.2622	
N752TL	0.2688			0.0522			-0.313			0.4558	
N515TL	0.2585			0.1887			-0.311			0.1826	
N161TL	0.2567			-0.2422			-0.2238			-0.1996	
N459TL	0.2555			0.1973			-0.2529			0.6466	
N166TL	0.2500			0.0382			-0.3751			0.2813	
N742TL	0.2393			-0.0269			-0.0091			0.015	
N607TL	0.2374			-0.0903			-0.0942			-0.1037	
N682TL	0.2364			-0.2217			-0.3619			-0.0121	
N477TL	0.2345			0.1763			-0.359			0.2148	
N635TL	0.2276			-0.2734	Deletion	Whole	-0.6251			-0.2229	
N524TL	0.2171			0.5084			-0.171			0.7496	
N566TL	0.2113			-0.0713			-0.4947			0.0817	
N614TL	0.2092			0.2882			-0.4678			0.397	
N462TL	0.2085			-0.2546			-0.4962			0.1667	
N085TL	0.2038			-0.1633			-0.2222			0.062	
N097TL	0.2000			0.1468			-0.046			0.2308	
N596TL	0.1966			0.0926			-0.4646			0.4488	
N482TL	0.1869			-0.245			-0.0386			0.0044	
N743TL	0.1864			-0.1897			-0.222			0.093	
N544TL	0.1657			-0.0244			-0.4145			0.4503	
N665TL	0.1651			0.1917			-0.2019			0.1383	
N557TL	0.1608			0.0356			-0.2886			0.4597	
N511TL	0.1600			-0.1783			-0.1361			-0.2091	

N062TL	0.1509			0.0322			-0.4373			0.0937	
N712L	0.1497			0.0302			-0.0664			0.0225	
N725TL	0.1438			-0.2036			-0.408			-0.0991	
N225TL	0.1394			0.1919			-0.2873			0.2837	
N676TL	0.1336			0.1138			-0.2759			0.066	
N598TL	0.1290			0.1951			-0.2914			0.4993	
N528TL	0.1212			0.0122			-0.3522			0.3073	
N521TL	0.1129			-0.1116	Deletion	Whole	-0.5155			0.2273	OGG1 missense variant (NP_058434.1: p.Met330Val)
N735TL	0.1128			0.2464			-0.1837			0.6641	
N685TL	0.1089			-0.1894			-0.1575			0.1499	
N205TL	0.0926			0.1145			-0.1274			0.3611	
N715TL	0.0896			0.2729			-0.4863			0.2347	
N569TL	0.0861			0.2332			0.0616			0.6667	
N655TL	0.0633			0.2436	Deletion	Whole	-0.5035			0.1802	
N523TL	0.0286			-0.0909			-0.114			0.1697	

Table S3: Substitutions in *MUTYH* knockout, *OGG1* knockout, *NUDT1* knockout, and wild-type clones

A) Normalized substitutions per genome

	Sample names clone/subclone pair	Days between clonal steps	Normalized substitutions per genome						
			C>A	C>G	C>T	T>A	T>C	T>G	All sub
MUTYH KO1	10/10.3	122	1546.941	108.6844	274.1262	241.5208	519.2698	107.4768	2798.019
MUTYH KO2	18/18.2	122	1400.637	103.4426	272.2801	186.6724	405.4476	84.41871	2452.898
OGG1 KO1	14/14.2	122	1446.434	94.17515	303.0508	235.4379	511.9265	103.8341	2694.858
OGG1 KO2	61/61.1	122	668.4098	68.03457	244.6857	144.4243	373.5933	68.03457	1567.182
NUDT1 KO1	4/4.1	122	231.9896	45.17693	136.7518	102.5638	285.7136	42.73494	844.9307
NUDT1 KO2	20/20.3	122	516.9151	151.9635	295.551	201.0225	471.4457	98.11814	1735.016
Wild-type 1	wt9/wt9.1	80	286.3941	60.48841	162.9484	130.8525	269.1117	41.97155	951.7666
Wild-type 2	wt1/wt1.2.1	126	394.315	103.9558	249.7328	200.7422	509.0248	92.00683	1549.777

B) Normalized substitutions per genome per day

	Sample names clone/subclone pair	Days between clonal steps	Normalized substitutions per genome per day						
			C>A	C>G	C>T	T>A	T>C	T>G	All sub
MUTYH KO1	10/10.3	122	12.67984	0.890856	2.246936	1.979679	4.25631	0.880957	22.93458
MUTYH KO2	18/18.2	122	11.48063	0.84789	2.231804	1.530101	3.323341	0.691957	20.10573
OGG1 KO1	14/14.2	122	11.85601	0.771927	2.484023	1.929819	4.196119	0.8511	22.089
OGG1 KO2	61/61.1	122	5.478769	0.55766	2.005621	1.183805	3.06224	0.55766	12.84576
NUDT1 KO1	4/4.1	122	1.901554	0.370303	1.120916	0.840687	2.341914	0.350286	6.925662
NUDT1 KO2	20/20.3	122	4.237009	1.245602	2.422549	1.647726	3.864309	0.804247	14.22144
Wild-type 1	wt9/wt9.1	80	3.579926	0.756105	2.036855	1.635656	3.363896	0.524644	11.89708
Wild-type 2	wt1/wt1.2.1	126	3.129484	0.825046	1.982007	1.593192	4.039879	0.730213	12.29982

C) Relative substitutions per genome per day

	Sample names clone/subclone pair	Days between clonal steps	Relative substitutions per genome per day						
			C>A	C>G	C>T	T>A	T>C	T>G	All sub
MUTYH KO1	10/10.3	122	0.55287	0.038843	0.097972	0.086319	0.185585	0.038412	1
MUTYH KO2	18/18.2	122	0.571013	0.042172	0.111003	0.076103	0.165293	0.034416	1
OGG1 KO1	14/14.2	122	0.536738	0.034946	0.112455	0.087366	0.189964	0.03853	1
OGG1 KO2	61/61.1	122	0.426504	0.043412	0.156131	0.092155	0.238385	0.043412	1
NUDT1 KO1	4/4.1	122	0.274566	0.053468	0.16185	0.121387	0.33815	0.050578	1
NUDT1 KO2	20/20.3	122	0.297931	0.087586	0.170345	0.115862	0.271724	0.056552	1
Wild-type 1	wt9/wt9.1	80	0.300908	0.063554	0.171206	0.137484	0.28275	0.044099	1
Wild-type 2	wt1/wt1.2.1	126	0.254433	0.067078	0.161141	0.12953	0.32845	0.059368	1

Table S4 Compound libraries used in this study

A) Compounds in standard compound library

(+)-JQ-1	Dexametasone	LY3023414	Romidepsin
6-Mercaptopurine	Dovitinib	LY3039478	Rucaparib phosphate
6-Thioguanine	Doxorubicin hydrochloride	Maphosphamide	Ruxolitinib
Abemaciclib	EHT 1864 (2HCL)	Masitinib	S63845
Actinomycin D	Ensartinib	Melphalan	Sapitinib
Afatinib	Entinostat	Merestinib	SAR405
Alectinib	Entospletinib	Methotrexate	Saracatinib
Alisertib	Entrectinib	Miransertib	Savolitinib
AMG 337	Epidaza	Mitoxantrone dihydrochloride	Selinexor
Apatinib mesylate	Erlotinib hydrochloride	MK-2206 dihydrochloride	Selumetinib
AT-406	Etoposide	MLN 4924	SHP099 hydrochloride
AT7519	Everolimus	MM-102	Sirolimus
Axitinib	EW-7197	Momelotinib	Sonidegib
AZD1775	Fenretinide	Mubritinib	Sorafenib
AZD4547	Galunisertib	MX69	Sunitinib
AZD5582	Ganetespib	Navitoclax	Talazoparib
AZD6738	Gemcitabine	Neratinib	Tanespimycin
AZD8055	Glasdegib	Nilotinib	Taselisib
BCT-100	GSK J4 hydrochloride	Niraparib	Tazemetostat
BIBR 1532	GSK1070916	Olaparib	Temozolomide
Binimetinib	GSK2636771	OTX-015	Temsirolimus
Birinapant	GSK269962A	Oxaliplatin	TH1579
Bortezomib	GSK525762	Paclitaxel	ThioTEPA
Buparlisib	I-BRD9	Palbociclib	Tivantinib
Busulfan	Ibrutinib	Panobinostat	Tofacitinib citrate
Cabozantinib S-malate	Icotinib	Pazopanib	Topotecan hydrochloride
Camptothecin	Idasanutlin	PCI-34051	Trametinib
Capmatinib	Imatinib mesylate	PD-0325901	Vandetanib
CC122	Ipatasertib	PD-1/PD-L1 inhibitor 1	Varlitinib
Cediranib	Iphosphamide	Perifosine	VE-822
Ceritinib	IPI549	Pexidartinib	Vemurafenib
Cisplatin	Irinotecan	PF-06651600	Venetoclax
Cobimetinib	Isotretinoin	Pictilisib	Vinblastine
CPI-455	KU-55933	Pinometostat	Vincristine sulfate
Crenolanib	KU-60019	Plerixafor	Vismodegib
Crizotinib	Lapatinib	Ponatinib	Vistusertib
CUDC-907	Larotrectinib sulfate	Prednisolone	Volasertib
Cytarabine	Lenvatinib	Prexasertib	Vorinostat
Dabrafenib	LGK974	Quizartinib	XAV-939
Dasatinib	Linsitinib	Ravoxertinib	YM155
Daunorubicin hydrochloride	LMK-235	Regorafenib	YO-01027
Decitabine	Lorlatinib	RG6146	
Defactinib	LTURM34	Ribociclib	

B) Compounds in DNA damage compound library

4SC-202 (free base)	DBeQ	Lexibulin	Rucaparib (phosphate)
5 P 22077	Decitabine	LFM-A13	RVX-208
5-Azacytidine	Dinaciclib	M344	SB-743921
5-Fluorouracil	DMAT	Melphalan	SBE13 (Hydrochloride)
6-Thioguanine	Droxinostat	Miriplatin	SCR7
A-966492	Ellagic acid	Mitomycin C	Scriptaid
ACY-1215	ELR510444	Mitoxantrone (dihydrochloride)	SGC0946
Alisertib	ENMD-2076	ML324	SJB2-043
Altretamine	ENMD-2076 (Tartrate)	MLN8054	SLx-2119
AMG 900	Entinostat	Mocetinostat	SN-38
AMG 925	Epirubicin (hydrochloride)	Mps1-IN-1	SNS-314
AMI-1	Epothilone D	Nelarabine	Sodium phenylbutyrate
Amodiaquin (dihydrochloride dihydrate)	Etoposide	NMS-873	SP2509
Amsacrine	ETP-46464	Nocodazole	SR-3677
ARRY-520 (R enantiomer)	Fasudil (Hydrochloride)	NU 7026	SRT 1720 (Hydrochloride)
AT7519 (trifluoroacetate)	Flavopiridol	NVP-HSP990	SRT 2104
AT9283	Flavopiridol (Hydrochloride)	Orotic acid	SU9516
Aurora A inhibitor	Folinic acid (calcium salt pentahydrate)	OTX-015	T0070907
AZ20	Fosbretabulin (disodium)	Oxolinic acid	TAK-901
AZD1152	FRAX486	Palbociclib (hydrochloride)	Teniposide
AZD1152-HQPA	G007-LK	Panobinostat	Teprenone
AZD-2461	Gemcitabine	Parthenolide	TG003
Balaglitazone	Gemcitabine (elaidate)	PF-03814735	TG-101348
b-AP15	GSK-1070916	PFI-1	Thio-TEPA
Belinostat	GSK2656157	PHA-767491 (hydrochloride)	THZ1 (Hydrochloride)
BG45	GSK343	PIK-75	Topotecan (Hydrochloride)
BIO	GSK3787	Pimelic Diphenylamide 106 (analog)	Tozasertib
BIX-01294	GSK-J1	Pirarubicin (Hydrochloride)	Treosulfan
BML-277	GSK-J2	PJ34 (hydrochloride)	Triapine
Bromosporine	GSK-J4	Poloxin	Trichostatin A
Capecitabine	GW 501516	PR-619	Triciribine
Carmofur	GW9662	Pracinostat	Triclabendazole
CCT241533 (hydrochloride)	Hesperadin	Pralatrexate	Troglitazone
CDK-IN-2	HMN-214	PRT4165	TTP 22
CHR-6494	Hydroxyfasudil (hydrochloride)	Purvalanol A	Tubastatin A (Hydrochloride)
CI-994	Ifosfamide	Purvalanol B	Tubastatin-A
Ciprofibrate	Inauhzin	PYR-41	UNC 0631
Clofarabine	IPA-3	PYZD-4409	UNC0379
Clofibrate	ISRIB (trans-isomer)	Remodelin (hydrobromide)	UNC1999
CP-466722	JIB-04	Resminostat (hydrochloride)	Valproic acid (sodium salt)
CVT-313	JW 55	RG2833	Veliparib (dihydrochloride)
CW-069	L-165041	RGFP966	Vinblastine (sulfate)
CX-5461	LDC000067	Rigosertib (sodium)	Vorinostat
D-64131	LDN-57444	RKI-1447	WAY-262611
Dacarbazine	LEE011	Ro3280	XL228
Dacinostat	LEE011 (succinate)	Ro-3306	XL413 (hydrochloride)
Danusertib	Levoleucovorin (Calcium)	Rosiglitazone (maleate)	ZM-447439
Daunorubicin (Hydrochloride)	Levomefolate (calcium)	Rosilitazone	β-Lapachone

Table S5: Genomic aberrations in RAS-MAPK pathway in relapse tumors from Eleveld et al. 2015 (11)

Nr	Patient ID	Gene	Genomic event	Type event	Cosmic ID	Detected in primary tumor	Type of mutation
2	FR_NB1269	ALK	Somatic mutation (L1196M)	activating	99137	Yes	C>A
3	FR_NB1382	ALK	Somatic mutation (Y1278S)	activating	28058	No	Other substitution
4	NL_N774	PTPN11	Somatic mutation (A72T)	activating	13014	Yes	Other substitution
5	US_PATNKP	FGFR1	Somatic mutation (N546K)	activating	19176	Yes	C>A
6	US_PASGAP	NF1	Somatic Mutation (Splice Donor) + Hemizygous Deletion	inactivating		No	C>A
7	NL_N790	ALK	Amplification and fusion	activating		No	Other mutation type
10	NL_N607	ALK	Somatic mutation (F1174L)	activating	28055	No	C>A
11	US_PARHAM	ALK	Somatic mutation (R1275Q)	activating	28056	Yes	Other substitution
12	US_PATYIL	NRAS	Somatic mutation (Q61K)	activating	580	Yes	C>A
13	NL_N571	NF1	Homozygous deletion	inactivating		No	Other mutation type
14	US_PASNPG	ALK	Somatic mutation (F1174I)	activating	28491	Yes	Other substitution
15	US_PARBAJ	HRAS	Somatic mutation (Q61K)	activating	496	No	C>A
17	FR_NB1224	ALK	Somatic mutation (R1275Q)	activating	28056	Yes	Other substitution
18	FR_NB0175	ALK	Somatic mutation (Y1278S)	activating	28058	Yes	Other substitution
19	FR_NB308	ALK	Somatic mutation (F1174L)	activating	28061	Yes	C>A
20	NL_N041	BRAF	Tandem duplication catalytic domain	activating		No	Other mutation type
21	US_PAPVEB	KRAS	Somatic mutation (G12D)	activating	521	Yes	Other substitution
23	FR_NB399	ALK	Somatic mutation (R1275Q)	activating	28056	Yes	Other substitution

Table S6 Sequences of gRNAs

	gRNA sequence (5’→3’)
MUTYH gRNA 1	AGGAAGCCACGAGCAGCCGT
MUTYH gRNA 2	GCATGCTAAGAACAACAGTC
OGG1 gRNA 1	GTACGATGCCCCATGCGCCT
OGG1 gRNA 2	AGTACGATGCCCCATGCGCC
NUDT1 gRNA 1	TTCGGGGCCGGCCGGTGGAA
NUDT1 gRNA 2	CCGGCCGGTGGAATGGCTTT

Table S7 Primer sequences for homology arm amplification. Underlined sequences are homologous to target vector

	Fwd primer (5’→3’)	Rev primer (5’→3’)
MUTYH 3’ homology arm 1	CGTGGGAAGTGGTCACAGGA	ACCCCCTTAAGCTTTGGAGC
MUTYH 5’ homology arm 1	ATAGGGAGAGCGGCCGCCAGAGGCTTAGCAGGGACTG	GAAGATCTGGCGGCCGCGCTGCTCGTGGCTTCCTCAT
MUTYH 3’ homology arm 2	GTCAGGCCAAGCCTTCTGCC	CACTTAGGGCTTCCCCCAAC
MUTYH 5’ homology arm 2	ATAGGGAGAGCGGCCGCCAGAGGCTTAGCAGGGACTG	GAAGATCTGGCGGCCGCTGTTGTTCTTAGCATGCTTC
OGG1 3’ homology arm 1 + 2	CGCATGGGGCATCGTACTCT	GAAGTGGGAGTCCACGGAAC
OGG1 5’ homology arm 1 + 2	ATAGGGAGAGCGGCCGCGAGGTCTCAGAGGACGCCTA	GAAGATCTGGCGGCCGCCCTGGGCAGAAGCGCGCG
NUDT1 3’ homology arm 1	GAATGGCTTTGGGGGCAAAG	TCGGAAAAGTAGAGCCCCCT
NUDT1 5’ homology arm 1	ATAGGGAGAGCGGCCGCACCTGGACCTGCACACCTAT	GAAGATCTGGCGGCCGCCACCGGCCGGCCCCGAAG
NUDT1 3’ homology arm 2	TTTGGGGGCAAAGTGCAAGA	GGCGGCTACTGCAACTTACT
NUDT1 5’ homology arm 2	ATAGGGAGAGCGGCCGCAAGGACCCTTGTGACTTCCAG	GAAGATCTGGCGGCCGCGCCATTCCACCGGCCGGC
PGK-Puromycin	TTCCGGATGGCTCGAGGAGACACGTTGTAAGGCTATAA	TGCTGAAAAACTCGAGCTCGAGGCCTGGGATCATAAC

Table S8 Primer sequences for genotyping of knockout clones.

	Primer sequence (5'→3')
PGK-GFP-Puro_rev	CTGCTAAAGCGCATGCTCCA
MUTYH_fwd	GAGGAGGGTAAGACAGTGCAAA
MUTYH_rev	ATACGTATCACAATCCCTTCCCA
OGG1_fwd	GAGGCCTCTGATGTCCTGAG
OGG1_rev	CCTACAATAGCTCCATGTACGA
NUDT1_fwd	CAGGAAGGACCCTTGTGACTT
NUDT1_rev	CAGAGGCTTCCAGATACGCC

Table S9 qRT-PCR primers used in this study

	Fwd primer sequence (5'→3')	Rev primer sequence (5'→3')
MUTYH	ACGCAGGAGGAATTTCACAC	GAGGACACCTGGGACCTTTT
OGG1	CATTGCCCAACGTGACTACA	CACAGGCTCCGGAAAAAGT
NUDT1	GGCCAGATCGTGTTTGAGTT	GAAGCAGGAGTGGAAACCAG
HPRT1	TGACACTGGCAAAACAATGCA	GGTCCTTTTCACCAGCAAGCT
UBC	ATTTGGGTCGCGGTTCTTG	TGCCTTGACATTCTCGATGGT

SI References

1. J. J. Molenaar *et al.*, Sequencing of neuroblastoma identifies chromothripsis and defects in neuritogenesis genes. *Nature* **483**, 589-593 (2012).
2. H. Gad *et al.*, MTH1 inhibition eradicates cancer by preventing sanitation of the dNTP pool. *Nature* **508**, 215-221 (2014).
3. J. Drost *et al.*, Use of CRISPR-modified human stem cell organoids to study the origin of mutational signatures in cancer. *Science* **358**, 234-238 (2017).
4. H. Li, R. Durbin, Fast and accurate long-read alignment with Burrows-Wheeler transform. *Bioinformatics* **26**, 589-595 (2010).
5. M. A. DePristo *et al.*, A framework for variation discovery and genotyping using next-generation DNA sequencing data. *Nat Genet* **43**, 491-498 (2011).
6. F. Blokzijl, R. Janssen, R. Van Boxtel, E. Cuppen, MutationalPatterns: An integrative R package for studying patterns in base substitution catalogues. 10.1101/071761 (2016).
7. M. Jager *et al.*, Measuring mutation accumulation in single human adult stem cells by whole-genome sequencing of organoid cultures. *Nat Protoc* **13**, 59-78 (2018).
8. F. Blokzijl, R. Janssen, R. van Boxtel, E. Cuppen, MutationalPatterns: comprehensive genome-wide analysis of mutational processes. *Genome Med* **10**, 33 (2018).
9. L. B. Alexandrov *et al.*, Signatures of mutational processes in human cancer. *Nature* **500**, 415-421 (2013).
10. A. Viel *et al.*, A Specific Mutational Signature Associated with DNA 8-Oxoguanine Persistence in MUTYH-defective Colorectal Cancer. *EBioMedicine* **20**, 39-49 (2017)
11. T. F. Eleveld *et al.*, Relapsed neuroblastomas show frequent RAS-MAPK pathway mutations. *Nat Genet* **47**, 864-871 (2015).
